# Langmuir Monolayers as Effective Models of Apical Epithelial Cell Membranes: Studies on the Effects of Phthalate Incorporation

**DOI:** 10.1021/acs.langmuir.6c01119

**Published:** 2026-04-28

**Authors:** Marcin Broniatowski, Paweł Wydro

**Affiliations:** † Department of Environmental Chemistry, Faculty of Chemistry, 201871 The Jagiellonian University in Kraków, ul. Gronostajowa 2, 30-387 Kraków, Poland; ‡ Department of Physical Chemistry and Electrochemistry, Faculty of Chemistry, 37799 The Jagiellonian University in Kraków, ul. Gronostajowa 2, 30-387 Kraków, Poland

## Abstract

Phthalates are the most common plasticizers on the market, produced in megaton quantities. Phthalates can be released from plastics and enter the human body, primarily through ingestion or inhalation. The phthalate used in the largest quantities is di­(2-ethylhexyl) phthalate, DEHP. However, this compound is toxic: mutagenic, carcinogenic, and endocrine-disrupting. Therefore, DEHP is gradually being replaced by other phthalates, primarily diesters with longer, unbranched, or iso-branched chains. Upon entering the human body, phthalates first interact with epithelial cells, primarily their apical membrane. Our research aimed to develop models of apical membranes and to examine the effects of DEHP and its substitutes on their physical properties. In the research, the fact that the apical membrane contains raft and nonraft regions was considered, so two different membrane models were created and studied. Lipid Langmuir monolayers were used as membrane models. The effects of phthalate incorporation on the mechanical properties of these membranes, their morphology (Brewster angle microscopy), and their crystal structure (grazing incidence X-ray diffraction) were studied. Studies have shown that phthalates primarily disrupt the raft regions of the apical membrane. It was demonstrated that replacing DEHP with phthalates with long, unbranched chains is a poor option because their impact on the model membranes is more unfavorable than DEHP itself. However, using phthalates with iso-branched chains seems reasonable. These compounds have a lesser effect on the properties of the model membranes, and given their significantly lower toxicity, their use seems to be a suitable direction for the development of plasticizer chemistry.

## Graphical abstract

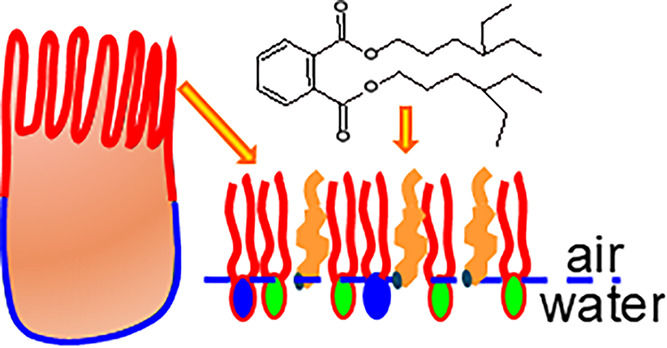

## Introduction

Phthalates, or *o*-phthalic acid esters, are synthesized in megaton quantities and used as plasticizers in many different plastics.^1,2^ Phthalates are so-called external plasticizers, meaning they are not covalently bonded to the polymer matrix, and thus can relatively easily migrate from it into the environment.^3^ The release of phthalates into the environment is facilitated by plastic degradation, particularly fragmentation, leading to the formation of microplastic particles.^4,5^ Organisms absorb phthalates from the environment through three main routes of exposure: dermal, ingestion, and inhalation.^6^ Phthalates absorbed from the environment can have a variety of toxic effects. Phthalates harm the reproduction of various organisms, disrupt the development of reproductive systems, and exposure to these substances may lead to infertility.^7–9^ Links between carcinogenesis and phthalate exposure are also widely reported, and some phthalates, such as di­(ethylhexyl) phthalate (DEHP), are classified as class II carcinogens.^10,11^ Phthalates absorbed via inhalation can also damage the lungs^12^ and lead to the development of asthma,^13^ and in aquatic organisms, cause gill damage.^14^ The cells that first come into contact with phthalate molecules after they enter the body, both through ingestion and inhalation, are epithelial cells. Epithelial damage caused by organic pollutants, including phthalates, can initiate oncogenesis or various inflammatory processes.^15–18^ Phthalates exhibit surface activity, limited water solubility, and significant affinity for lipids.^19–21^ Therefore, phthalates can penetrate cell membranes and, by accumulating within them, affect their basic functions, such as fluidity and permeability.^20,22,23^

DEHP is the main plasticizer in PVC, which is used to make bags for storing blood and blood-derived products. Therefore, DEHP was the first to be observed to incorporate into erythrocyte membranes, thereby significantly affecting their morphology.^24,25^ Interestingly, DEHP incorporation into erythrocyte membranes was initially assumed to be beneficial, increasing the durability of blood products.^25^ However, over time, it was realized that the membrane activity of phthalates may be the cause of their ecotoxicity to a wide range of organisms. Phthalates disrupt the function of bacterial membranes,^26^ plasma and thylakoid membranes in algae^27^ and plants,^28^ and plasma and mitochondrial membranes in aquatic animals.^29^ Worse still, replacing phthalates with nonphthalate plasticizers, such as organic phosphoric acid triesters, citric acid triesters, or adipic acid diesters, did not solve the ecotoxicity problem, as these compounds also exhibit a strong tendency to damage membranes.^30,31^ Therefore, awareness of the toxicity and technological limitations of nonphthalate plasticizers is intensifying the search for DEHP substitutes among *o*-phthalic acid diesters. Current research indicates that membrane damage caused by phthalates may be related to their selective incorporation into lipid rafts and disruption of these structures, so crucial for proper cellular function.^32^ The transformation of epithelial cells into cancer cells is also increasingly associated with lipid raft damage.^33–35^

After entering the animal body, phthalates are absorbed from the gastrointestinal tract or from the respiratory tract by epithelial cells. Epithelial cells are highly polarized, and their membranes vary significantly in function and composition depending on the cellular region.^36,37^ The apical portion of the cell faces the outside world, the lumen of the organ (e.g., the intestinal lumen, renal tubules, or airways). This portion of the cell has a large specific surface area due to its extensive folding and numerous villi.^38,39^ The part of the membrane facing other epithelial cells is the basolateral part. The basolateral part is separated from the apical part by the intercellular junction, which ensures the maintenance of distinct lipid compositions between the two parts.^40^ The apical membrane of epithelial cells is rich in sphingolipids and cholesterol, while the composition of the basolateral membrane more closely resembles that of other nonpolar animal cells.^36,41–43^ Apical membranes are particularly rich in lipid rafts,^44–46^ which coalesce into larger structures called raft regions.^47^ It is estimated that raft regions, rich in sphingolipids and cholesterol, occupy approximately 60% of the apical membrane surface.^47^ However, this membrane is highly mosaic, with raft regions separated by nonraft regions, less condensed, and more fluid.^48^

Whether phthalates are similarly incorporated into both regions of the apical membrane is unknown. It is also unknown whether phthalates disrupt the structure of both regions or whether they are selectively destructive of raft regions, which would be particularly unfavorable given the correlation between lipid rafts’ damage in epithelial membranes and carcinogenesis. Furthermore, it is unknown whether commercially used phthalate DEHP substitutes exhibit lower membrane activity and disrupt the structure and function of the apical membrane to a lesser extent. It is even possible that some DEHP substitutes are more destructive to lipid rafts than this primary plasticizer. Therefore, it is worthwhile to research this issue. Because native apical membranes are highly complex systems, we used lipid Langmuir monolayers as models of apical membranes. Such a model reduces the complexity of biological membranes to simplified systems; however, its use enables control of the composition and packing of lipids, which is often useful for explaining the mechanisms of interactions between the xenobiotic under study and membrane lipids.^49,50^ In the studies, both the raft and nonraft regions of the outer lamella of the apical membrane were modeled. The basic research questions that can be asked here are (1) Do phthalates incorporate into the model membranes? (2) Are there noticeable differences in the interactions of phthalates with models of raft and nonraft regions of the apical membrane? (3) Whether and to what extent do they influence their physical properties and morphology? (4) Does the effect of phthalates depend on the structure of their aliphatic chains? Finding answers to these questions will lead to a better understanding of the mechanism of phthalate toxicity, particularly the initial stages related to the damage of apical membranes caused by these xenobiotics. Researching a broad set of phthalates can aid in the rational selection of new, less toxic plasticizers and contribute to reducing the risks associated with plastic use.

## Experimental Section

### Chemicals

All lipids used in our studies: cholesterol, sphingomyelins: chicken egg sphingomyelin (egg-SM) and bovine milk sphingomyelin (milk-SM), 1,2-dipalmitoyl-*sn*-glycero-3-phosphocholine (DPPC), and 1-palmitoyl-2-oleoyl-*sn*-glycero-3-phosphocholine (POPC) were purchased from Avanti Polar Lipids as lyophilized powders of the 99% purity. All the studied phthalates: di­(2-ethylhexyl) phthalate (DEHP), di-*n*-octyl phthalate (DOP), diisodecyl phthalate (DIDP), di-*n*-decyl phthalate (DDP), di-*n*-tridecyl phthalate (DTP), and cyclohexane-1,2-dicarboxylic acid diisononyl ester (DINCH) were purchased from Merck Sigma-Aldrich as analytical standards of the 99% purity. Spectroscopic-grade chloroform (>99.5% purity) stabilized by ethanol and spectroscopic-grade methanol (>99.5% purity) were purchased from Merck Sigma-Aldrich. Ultrapure water of the 18.2 MΩ•cm resistivity was produced in our laboratory using the Milli-Q Synergy 12 water purification system.

### Solutions

Samples of the studied lipids and phthalates were weighed on a Mettler-Toledo analytical balance with an accuracy of 10 μg and dissolved in the chloroform/methanol 9/1 v/v mixture in 10 mL class A glass volumetric flasks. The concentrations of the lipid solutions varied from 0.2 to 0.3 mg/mL, and those of phthalates from 0.1 to 0.2 mg/mL. All the stock solutions were stored at −20 °C. The ternary lipid mixtures imitating the outer lamellae of the epithelium apical membrane were prepared by mixing appropriate volumes of the lipid stock solutions in 5 mL glass volumetric flasks. The lipid solutions doped with phthalates were prepared in amber glass vials by mixing appropriate volumes of the ternary lipid mixtures with a stock solution of a given phthalate just before the experiment. The studied mole ratios of phthalates were 0.05, 0.10, 0.15, and 0.20.

### Models of the Outer Lamellae of the Epithelium Apical Membrane

According to numerous publications, lipid rafts are an extremely important structure in the apical membrane of epithelia.^44–46^ Naturally, rafts, as structures composed of both lipids and various transmembrane proteins, occur throughout the entire membrane (both lamellae).^51,52^ However, higher concentrations of “raft lipids” are particularly prevalent in the outer lamella.^53^ According to available knowledge, lipid rafts found in the outer lamella of the apical membrane aggregate, forming large (compared to a single raft) raft regions.^47^ There are also nonraft regions in the outer lamella of the apical membrane, characterized by greater fluidity and lower content of “raft lipids”.^48^ Our goal was to reproduce the lipid matrices of both these regions as faithfully as possible, develop models, and use them to study the effect of selected plasticizers on their structure and physical properties. According to the scientific literature, a model lipid raft should certainly contain sphingomyelin and cholesterol, and the typical molar ratio of sphingolipids, or phospholipids in general, to cholesterol is 2:1.^53^ On the other hand, the scientific literature demonstrates that in the apical membrane, sphingomyelin is certainly accompanied by phosphatidylcholines.^54,55^ Therefore, the models were ternary systems composed of sphingomyelin, phosphatidylcholine, and cholesterol. For the raft region model, the 2:1 phospholipid/cholesterol ratio was maintained; for the nonraft region model, the phospholipid/cholesterol ratio was increased to 3:1. The lipid raft model should be characterized by significant condensation,^51^ so saturated phosphatidylcholine (DPPC) was used as its component. The nonraft region model should be more expanded, hence mixed-chained PC (POPC) was used. The key question is which sphingomyelins to use to create these models. Sphingomyelins of the outer lamella of cell membranes typically contain a saturated fatty acid chain, but they can vary in chain length.^56–58^ For the studies, two natural sphingomyelins extracted from chicken eggs and bovine milk were purchased. Single-component Langmuir monolayers on a pure aqueous subphase were formed and characterized. π-A isotherms and *C*_S_^–1^-π curves can be found in Figure S1 in the Supporting Information. It was found that egg-SM forms much more condensed monolayers than milk-SM, even though milk-SM contains more long-chain fatty acids than egg-SM.^56,58^ The reason for this may be the small (up to 10%) share of monounsaturated fatty acids, such as 18:1 or 24:1, in the fatty acid profile of milk-SM.^56–58^ Therefore, egg-SM was used to model lipid rafts and milk-SM to model nonraft regions. According to the scientific literature, the SM:PC molar ratio in the apical membrane can be approximately 2:1.^43,59^ This ratio was maintained when modeling the raft regions. For nonraft regions, the SM:PC molar ratio of 1:1 was assumed. The molar ratios and compositions of both models are summarized in Table 1.

**Table 1:** Composition of the Studied Membrane Models

Model	Composition	Molar proportion
Raft (Mod1)	Egg-SM/DPPC/cholesterol	0.45/0.22/0.33
Nonraft (Mod2)	Milk-SM/POPC/cholesterol	0.375/0.375/0.25

### Langmuir Studies

In our studies, we used three different Langmuir troughs. Basic surface pressure (π)- mean molecular area (A), π-*A* isotherm measurements were performed on a KSV-NIMA (Biolin Scientific, Sweden) double-barrier trough (area 273 cm^2^). Monolayers for the BAM experiments were compressed on a larger KSV-NIMA double-barrier instrument of an area of 580 cm^2^. A custom-made single-barrier R&K Langmuir trough of the area of 500 cm^2^ was installed in the Sirius beamline of the SOLEIL synchrotron. After each experiment, the Teflon troughs of the instruments were wiped with a tissue soaked in chloroform, followed by a tissue soaked in isopropanol, and rinsed with plenty of Milli-Q water. In all the experiments, Milli-Q water was used as a subphase. An appropriate volume of lipid or lipid + phthalate solution was deposited at the air/water interface using Hamilton analytical syringes. Ten minutes were left for the spreading solvent evaporation, after which the monolayers were compressed at a constant compression rate of 10 cm^2^·min^–1^. Surface pressure was monitored using a Wilhelmy-type electrobalance (KSV NIMA) with a rectangular filtration paper plate (Whatmann, ashless) as the surface pressure sensor. The accuracy of π measurements was ± 0.05 mN/m. All the experiments were repeated at least three times. The uncertainty of A was ± 1 Å^2^/molecule. All the measurements were performed at a constant subphase temperature of 20 ± 0.1 °C using a water-circulating bath (Julabo). To discuss the degree of the monolayers’ condensation and to comment on their physical state, compression modulus *C*_S_^–1^ was calculated from the course of the π-*A* isotherms following its definition^60^

1 $$C_{S}^{-1}=-A{\left(\frac{d\pi }{dA}\right)}_{T}$$

The studied phthalates are surface active and form Langmuir monolayers when spread at the air/water interface from chloroform solution.^20^ Thus, for the systems containing the studied lipids and phthalates, it was possible to calculate thermodynamic excess functions of mixing: the excess free energy of mixing Δ*G* ^exc^ and the excess area of mixing *A* ^exc^. These functions are defined as follows^61^

2 $${\Delta G}^{\mathrm{exc}}=N_{A}\int_{0}^{\pi }A^{\mathrm{exc}}d\pi$$

Where *N*_A_ is Avogadro number

3 $$A^{\mathrm{exc}}=A_{12}-A_{\mathrm{id}}$$

Where: *A*_12_ is the mean molecular area observed for a two-component monolayer made of molecules 1 and 2 at a given π value. *A*_id_ is the mean molecular area calculated for the same two-component monolayer assuming ideal mixing of the components

4 $$A_{\mathrm{id}}=A_{1}X_{1}+A_{2}X_{2}$$

Where: *A*_1_—mean molecular area observed for the one-component monolayer made of molecule 1 at the same π value. *X*_1_ is the molar ratio of substance 1 in the mixture. *A*_2_ and *X*_2_ are defined identically for substance 2.

### Brewster Angle Microscopy

A Brewster angle microscope—UltraBAM (Accurion GmbH, Goettingen, Germany) equipped with a 50 mW laser emitting p-polarized light at a wavelength of 658 nm, a 10× magnification objective, polarizer, analyzer, and a CCD camera was used. The spatial resolution of the microscope was 2 μm. The foregoing apparatus and the Langmuir trough were placed on a table (Standa Ltd., Vilnius, Lithuania) equipped with an active vibration isolation system (antivibration system VarioBasic 40, Halcyonics, Göttingen, Germany).

All the BAM experiments were repeated at least twice, each on a newly prepared monolayer. If there were noticeable differences in the morphology of the studied monolayers, a third, decisive experiment was performed.

### Grazing Incidence X-ray Diffraction

GIXD experiments were performed on the Sirius beamline of the SOLEIL synchrotron (Gif sur Yvette, France) using a dedicated surface diffractometer.^62,63^ The description of the settings of this instrument and the performance of a routine experiment can be found in the experimental section of reference.^64^ All the GIXD experiments were repeated twice. Theoretical introduction to the GIXD technique can be found in the publications of its pioneers, K Kjaer and J Als-Nielsen.^65,66^

## Results and Discussion

### Physicochemical and Structural Characterization of the Model Systems

Monolayers mimicking the lipid matrix of the raft regions (Model1, Mod1) and nonraft regions (Model2, Mod2) of the outer lamella of the apical membrane of epithelial cells were studied on Langmuir trough. The results of these studies, along with the basic results obtained using the GIXD technique, are presented in Figure 1. π-*A* isotherms, *C*_S_^–1^-π curves, and Bragg peak plots for one-component lipid monolayers formed of the models’ components can be found in Figures S1 to S3 in the Supporting Information.

**Figure 1:**
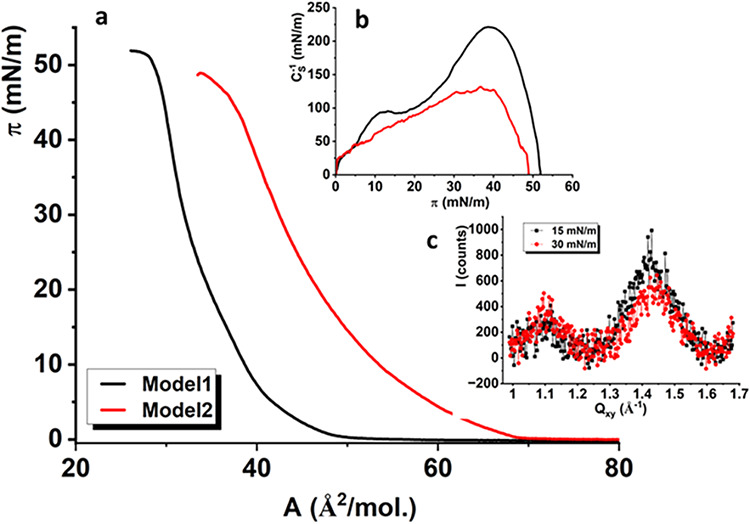
Characterization of the model membranes: (a) π-*A* isotherms, (b) *C*_S_^–1^-π curves, (c) *I*(*Q*_*xy*_) Bragg peak plots.

According to the original assumption, the models of raft (Model 1) and nonraft (Model 2) regions differ significantly in the degree of condensation, which is already reflected in the value of the *A*_0_ parameter, i.e., the mean molecular area at which the surface pressure begins to increase during monolayer compression: 47.4 Å^2^/mol. and 68.7 Å^2^/mol., respectively.

In the course of the π-*A* isotherm of the Mod1 monolayer, no plateau region can be observed, but at a pressure of about 15 mN/m, a clear inflection point occurs in the curve. Before this point, the surface pressure increases relatively slowly; after it, it increases much faster. The monolayer collapses at an area of about 28 Å^2^/mol and π = 51.4 mN/m. Up to the surface pressure corresponding to the mentioned inflection point in the isotherm, the compression modulus values do not exceed 100 mN/m. The inflection point of the isotherm manifests itself as a shallow minimum in the *C*_S_^–1^-π curve. A sharp increase follows this minimum in the compressibility modulus, which reaches the maximum value of 220 mN/m. The flat region extending from π 10 to 20 mN/m in the *C*_S_^–1^-π curve corresponds to an equilibrium in the monolayer between the liquid expanded (LE) and the liquid condensed (LC) states; above π = 20 mN/m the monolayer state can be defined as LC.

During compression of the Mod2 monolayer, the surface pressure increases relatively slowly and steadily, with no discernible plateau or inflection point in the curve. The monolayer collapses at an area of 36.5 Å^2^/mol. and π = 46.7 mN/m. During compression of the monolayer, the compressibility modulus increases almost linearly, reaching 100 mN/m at π = 23 mN/m. For larger values of π, *C*_S_^–1^ slightly exceeds 100 mN/m, reaching a maximum value of 130 mN/m. Therefore, it can be concluded that the original assumption was achieved—the model of nonraft regions is characterized by limited condensation, and based on the compressibility modulus criterion, the Mod2 monolayer remains in the LE state up to the collapse pressure.

Both model membranes were compressed to a surface pressure of 15 mN/m and then, in an independent experiment, to 30 mN/m, after which the π value was stabilized at a given level, and the monolayers were examined using the GIXD technique. These studies, however, were preceded by GIXD measurements for one-component monolayers formed from lipids that were components of the studied models. Cholesterol, a component of both models, forms two-dimensional crystalline domains in its monolayers, diffracting the synchrotron beam. The Bragg peak for cholesterol is characterized by significant intensity but also a large half-width, indicating a small extent of crystallinity in the monolayer plane^67^—Figure S3c. For cholesterol, the courses of the *I*(*Q*_*xy*_) curves are identical within the error limits, regardless of whether the measurement was performed at π = 15 or 30 mN/m. This is due to the fact that at both pressure values, cholesterol molecules are aligned perpendicularly at the water/air interface, and their in-plane packing is described by a two-dimensional trigonal lattice.^68^

DPPC also diffracts the synchrotron beam intensely. Depending on the authors and the surface pressure at which the GIXD measurements for DPPC monolayers were performed, either three diffraction maxima—a two-dimensional monoclinic lattice or two maxima—a two-dimensional centered rectangular lattice, are distinguished.^69,70^ The location of the maxima and their intensity strongly depend on the surface pressure at which the measurement was performed. As pressure rises, the number of crystalline domains in the DPPC monolayer increases, leading to an intensity increase, while tilt, the angle between the principal axis of the hydrocarbon chains in the DPPC molecule and the normal to the monolayer, decreases. The tilt value and its azimuth determine the number and relative position of diffraction maxima.^65,66^ The Bragg peaks measured for DPPC are presented in Figure S3b.

Sphingomyelins, in turn, are characterized by a lower degree of packing than phosphatidylcholines with the same fatty acid chains.^71,72^ The literature primarily presents diffraction patterns of natural SM extracted from hen eggs, which is not a pure chemical compound but a mixture dominated (approximately 85%) by palmitic sphingomyelin. Ultimately, egg-SM scatters the synchrotron beam poorly, with very weak signal intensity compared to DPPC or cholesterol, and a very large Bragg peak width, indicating a very small extent of crystallinity in the monolayer plane, or, in other words, a very small diameter of crystalline nanodomains in the egg-SM monolayer.

The Bragg peaks for our egg-SM sample measured at 15 and 30 mN/m are shown in Figure S3a. At 15 mN/m, the diffraction signal is very weak, barely exceeding noise. At 30 mN/m, the signal is more intense and is a superposition of two distinct diffraction maxima.^71,72^ Milk-SM and POPC do not form crystalline monolayers at any surface pressure and therefore do not scatter synchrotron radiation.

Bragg peaks, i.e., the dependence of the intensity on the *Q*_*xy*_ component of the scattering vector for the Mod1 model, are shown in Figure 1c. First, the Mod1 monolayer diffracts the synchrotron radiation beam, which means that it can be concluded that 2D-crystalline nanodomains are present in it. The signal intensity is weak, but clearly above the noise. At a pressure of 30 mN/m, the signal for the Mod1 model has a lower intensity than that recorded for the single-component egg-SM monolayer. On the other hand, the signal recorded at 15 mN/m has a clearly higher intensity than that recorded for the single-component egg-SM monolayer. Due to the increased intensity of the diffraction signal at π = 15 mN/m, the GIXD technique could be used to study the effect of phthalate incorporation into the Mod1 monolayer on its 2D crystalline structure. In the diffraction pattern of the Mod1 model in Figure 1c, two weak diffraction maxima can actually be distinguished. A stronger one with the maximum intensity at *Q*_*xy*_ around 1.45 Å^–1^ and a much weaker one at *Q*_*xy*_ around 1.15 Å^–1^. The stronger maximum is the result of synchrotron beam diffraction on 2D crystalline domains mimicking a lipid raft, composed of DPPC, SM, and cholesterol. This maximum is located at the *Q*_*xy*_ value of 1.45 Å^–1^, typical for phospholipids; however, the very large half-width indicates the presence of cholesterol in these crystalline nanodomains.^73,74^ In the scientific literature on lipid raft modeling, two types of cholesterol are often distinguished: raft cholesterol and excess cholesterol.^75–77^ We used a phospholipid/cholesterol molar ratio of 2/1, which is typical for rafts. However, the GIXD results indicate that a small number of cholesterol molecules in our model are excess cholesterol, meaning they do not incorporate into ternary domains but form separate domains.

The evolution of the model membrane morphology during compression was also studied using BAM microscopy. The low-condensation model of the nonraft regions of the apical membrane (Mod2) was homogeneous from the beginning of the compression until the monolayer collapse. This behavior is typical for monolayers in the LE state.^78^ The situation was completely different for the model of the raft regions (Mod1), for which the evolution of the monolayer morphology was observed during compression. Therefore, the BAM technique was used in further studies only to image model 1. Representative BAM images obtained during compression of the Mod1 monolayer are shown in Figure 2.

**Figure 2:**
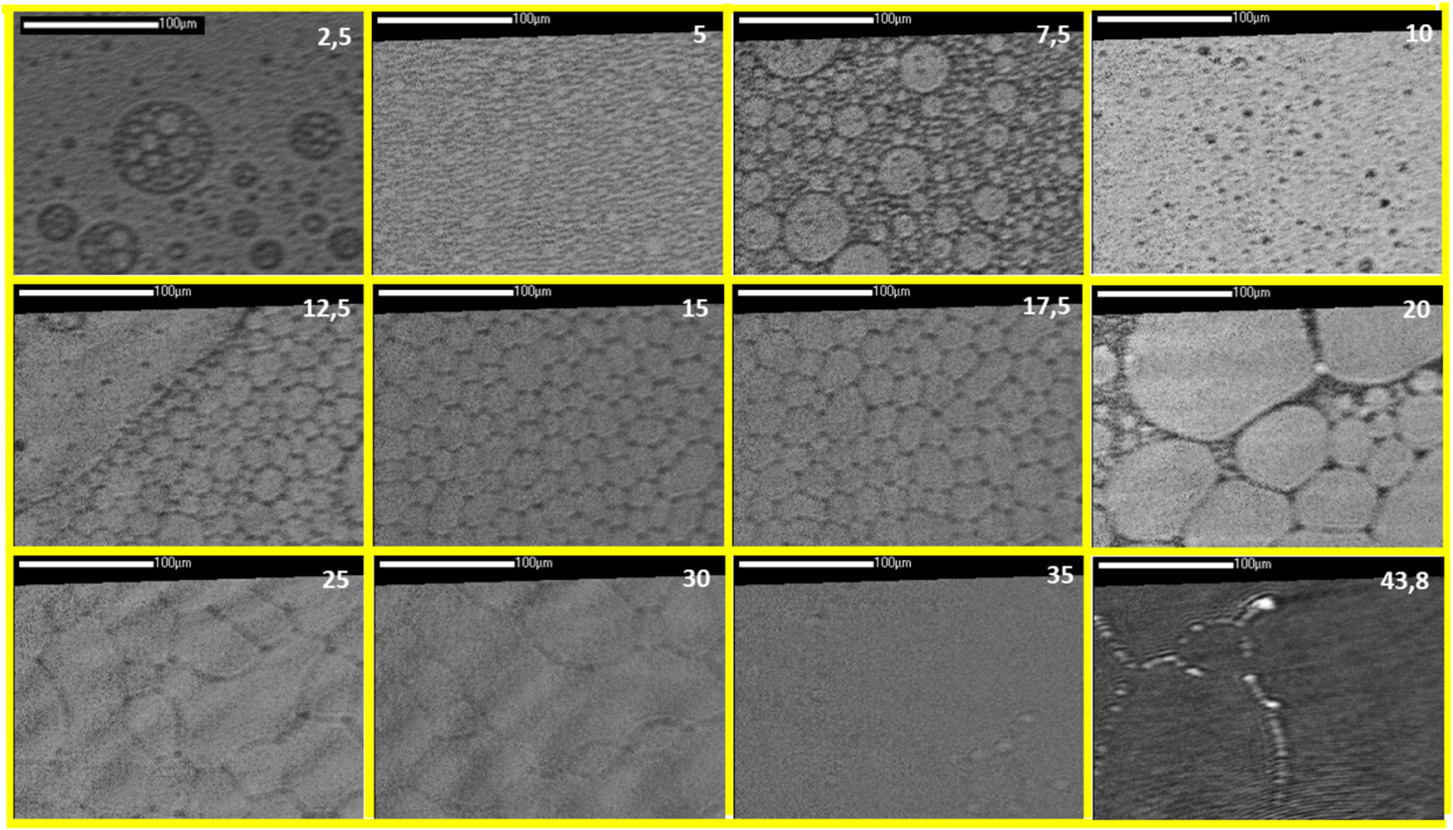
Representative BAM images for the Mod1 monolayer. The numbers in the pictures indicate surface pressure (mN/m). The scale bar indicates 100 μm.

At low surface pressures (2.5 mN/m in Figure 2), so-called foam structures were observed, typical of equilibria between the gaseous and liquid states of the expanded monolayer.^78^ However, already at π = 5 mN/m, the BAM image shows numerous small condensed domains, which, as seen in the next image (7.5 mN/m), merge and grow. The π value here is still below the inflection point of the π-A isotherm, which in the above description was identified as a manifestation of the LE-LC phase transition occurring in the monolayer. However, BAM measurements show that condensed domains form at lower π values. The image of the monolayer across its entire extent between the compressing barriers is not necessarily identical. The morphology of the monolayer evolves locally, and we see only a small fragment of it in the microscope’s field of view. Therefore, at π = 10 mN/m, small condensed domains were again observed, similarly to those observed at π = 5 mN/m. Images in the π range from 12.5 to 17.5 mN/m resembled the image first observed at 7.5 mN/m—numerous condensed domains with diameters of about 20 μm were in equilibrium with the monolayer in the LE state (darker background image between domains). At a pressure of 20 mN/m, further fusion of condensed domains occurred in the monolayer, forming very large monolayer domains in the LC state with diameters often exceeding 100 μm, but as shown by images recorded at π = 25 and 30 mN/m, full homogenization of the monolayer and complete disappearance of the LE phase were not yet achieved. Only at π = 35 mN/m was the Mod1 monolayer homogeneous, and the LE phase was no longer observed. The first multilayer aggregates were observed at a pressure of 44 mN/m, which is several mN/m lower than the collapse pressure of the monolayer.

### The Effect of DEHP on Apical Membrane Models

The study of the effect of phthalates on apical membrane models began with the most common plasticizer, DEHP. It should be noted at the outset that all the plasticizers studied here are surface-active and form Langmuir monolayers. We recently published isotherms for these monolayers.^20^ As a reminder, we present them in Figure S4 in the Supporting Information. Phthalates are not typical surfactants in that they lack a polar group that strongly anchors them at the water/air interface. Therefore, the collapse pressures of phthalate monolayers are low, in the range of 15–20 mN/m. This is important because at higher π values for mixed monolayers, phthalates can be expected to separate from lipid monolayers and form multilayer aggregates on the monolayer surface. Therefore, the BAM image sets in the main manuscript are shown at π = 15 mN/m. Similarly, the GIXD measurements were also performed at this surface pressure.

The π-A isotherms for the Mod1_DEHP and Mod2_DEHP systems are presented in Figure S5 in the Supporting Information, while in Figure S6A, the complete set of BAM images for the Mod1_DEHP system is presented, as well as images showing multilayer aggregates—Figure S6B. Here, we focus on the discussion of the *C*_S_^–1^-π curves and the Δ*G* ^exc^–*X*(DEHP) dependences. These plots, together with the BAM images recorded at π = 15 mN/m, are presented in Figure 3.

**Figure 3:**
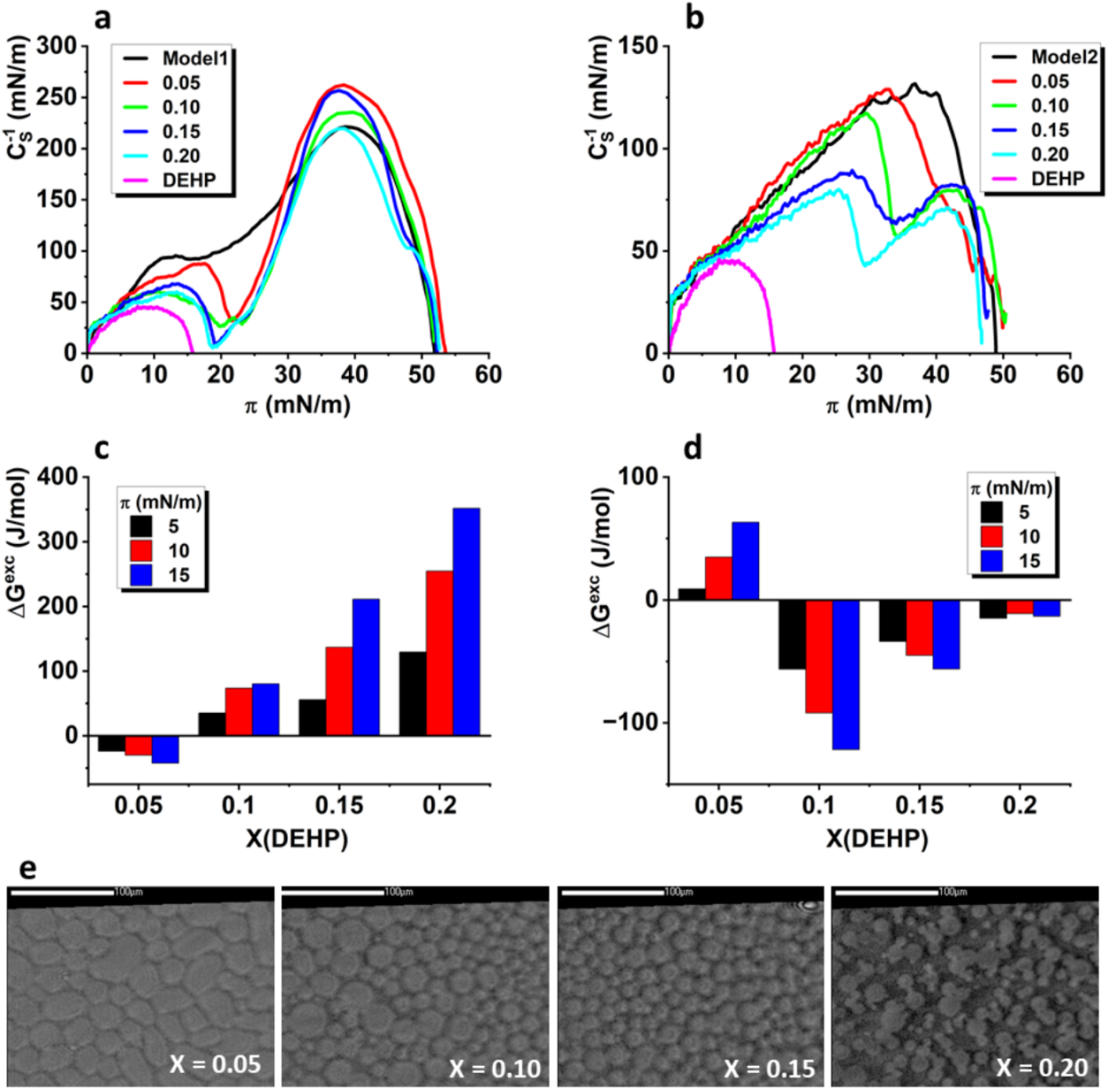
Results for the model membranes enriched in DEHP. (a, b) *C*_S_^–1^-π curves, (c, d) Δ*G* ^exc^–*X*(DEHP) dependences for the systems: (c) Mod1_DEHP, (d) Mod2_DEHP. (e) BAM images recorded at π = 15 mN/m for the Mod1 membrane enriched in DEHP. *X*(DEHP) is indicated in the images; the scale bar indicates 100 μm.

The introduction of DEHP into the Mod1 monolayer significantly changes the course of the *C*_S_^–1^-π curve. First, a deep minimum appears in the curve. The appearance of this minimum corresponds to the surface pressure value at which multilayer aggregates begin to appear at the water/air interface—Figure S6B, i.e., it is associated with the removal of phthalate molecules from the lipid monolayer. On the other hand, however, at any X­(DEHP), no such aggregates are observed up to π = 20 mN/m—Figure S6A. Two opposing trends can be observed in the *C*_S_^–1^-π curve. Before the minimum, increasing *X*(DEHP) leads to a systematic decrease in the compression modulus, i.e., the DEHP molecules hinder the nucleation and growth of condensed domains in the Mod1 monolayer. On the other hand, for *X*(DEHP) = 0.05, 0.10, and 0.15, a clear increase in the maximum *C*_S_^–1^ value is observed. This indicates that not all DEHP molecules are removed from the lipid monolayer during its compression. Some of them remain incorporated into the Mod1 monolayer up to the collapse pressure. These molecules cause an increase in monolayer condensation at high π values. The morphology of DEHP-enriched monolayers at π = 15 mN/m is very similar to that observed for the initial Mod1 monolayer. Only at *X*(DEHP) = 0.20 does the LE phase fraction clearly increase, i.e., for lower mole fractions, the effect of DEHP on the lipid monolayer, inferred from the *C*_S_^–1^-π expansion curves, is only weakly visible. As for the full set of BAM images (Figure S6A), the presence of DEHP somewhat unifies the size of the condensed domains, with no very fine domains being observed. Furthermore, at X­(DEHP) = 0.05 and 0.10 and π = 20 mN/m, the monolayer is almost homogeneous, which would confirm the organizing and condensing effect of DEHP at higher π values. However, observing this effect at much higher π values is not possible due to the formation of numerous multilayer aggregates.

For the Mod2_DEHP system, the situation is different. At X­(DEHP) = 0.05, the *C*_S_^–1^-π curve practically coincides with that recorded for the undoped Mod1 monolayer, meaning that at this molar ratio, DEHP has practically no effect on the condensation of the model membrane. Furthermore, at X­(DEHP) = 0.05, there is no minimum in the *C*_S_^–1^-π curve, meaning that DEHP molecules do not separate from the monolayer until the collapse pressure. For larger X­(DEHP), a minimum appears in the *C*_S_^–1^-π curve, but only for π > 30 mN/m, meaning that only at very high surface pressures do DEHP molecules separate from the monolayer. At *X*(DEHP) = 0.15 and 0.20, a clear reduction in the maximum *C*_S_^–1^ value is observed, i.e., a certain expansion effect.

Since phthalates are surface-active and form Langmuir monolayers, it was possible to calculate the excess free energy of mixing Δ*G* ^exc^ for the studied systems and plot them as a function of *X*(DEHP). For the Mod1_DEHP system, at *X*(DEHP) = 0.05, Δ*G* ^exc^ has a negative sign, and the values are close to 0 J/mol, whereas at *X*(DEHP) = 0.10, the function sign is positive, but the values are still very small, not exceeding 100 J/mol. It can therefore be concluded that at low contents, the miscibility of DEHP molecules with the lipids of the Mod1 monolayer is close to ideal. For *X*(DEHP) = 0.15 and 0.20, Δ*G* ^exc^ has a positive sign, and the values are clearly larger, indicating energetically unfavorable interactions between DEHP molecules and monolayer lipids. In the Mod2_DEHP system, on the other hand, the Δ*G* ^exc^ values are very close to 0 J/mol—positive at *X*(DEHP) = 0.05 and negative for the remaining mole ratios. Such a Δ*G* ^exc^–X­(DEHP) dependence for this system indicates virtually ideal miscibility of DEHP molecules with model 2 lipids.

### The Effect of DINCH and DOP on Apical Membrane Models

DINCH is the diisononyl ester of cyclohexane-1,2-dicarboxylic acid. It can be considered a derivative of the popular plasticizer diisononyl phthalate, in which the benzene ring has been completely hydrogenated to a cyclohexane ring. DINCH was introduced to the market around the year 2000 and has now completely replaced DEHP in many plastic products in Europe.^79–81^ From a toxicological perspective, it is usually perceived as a much less harmful substance than DEHP.^82^ *N*-octyl phthalate, DOP, has not found such widespread applications as a plasticizer.^83^ However, it is worth including this compound in the research. It is an isomer of DEHP, so using this compound will allow us to determine how replacing the branched 2-ethylhexyl chain with an unbranched *n*-octyl chain affects the interactions of phthalate with the model membrane.

The π-*A* isotherms for the Mod1_DINCH, Mod1_DOP, Mod2_DINCH, and Mod2_DOP systems are presented in Figure S7 in the Supporting Information. Full sets of BAM images can also be found there: for DINCH, Figure S8A,B, for DOP, Figure S9A,B. Figure 4 presents the *C*_S_^–1^-π plots for the systems discussed here, as well as the BAM images recorded for the Mod1_DINCH system at π = 15 mN/m.

**Figure 4:**
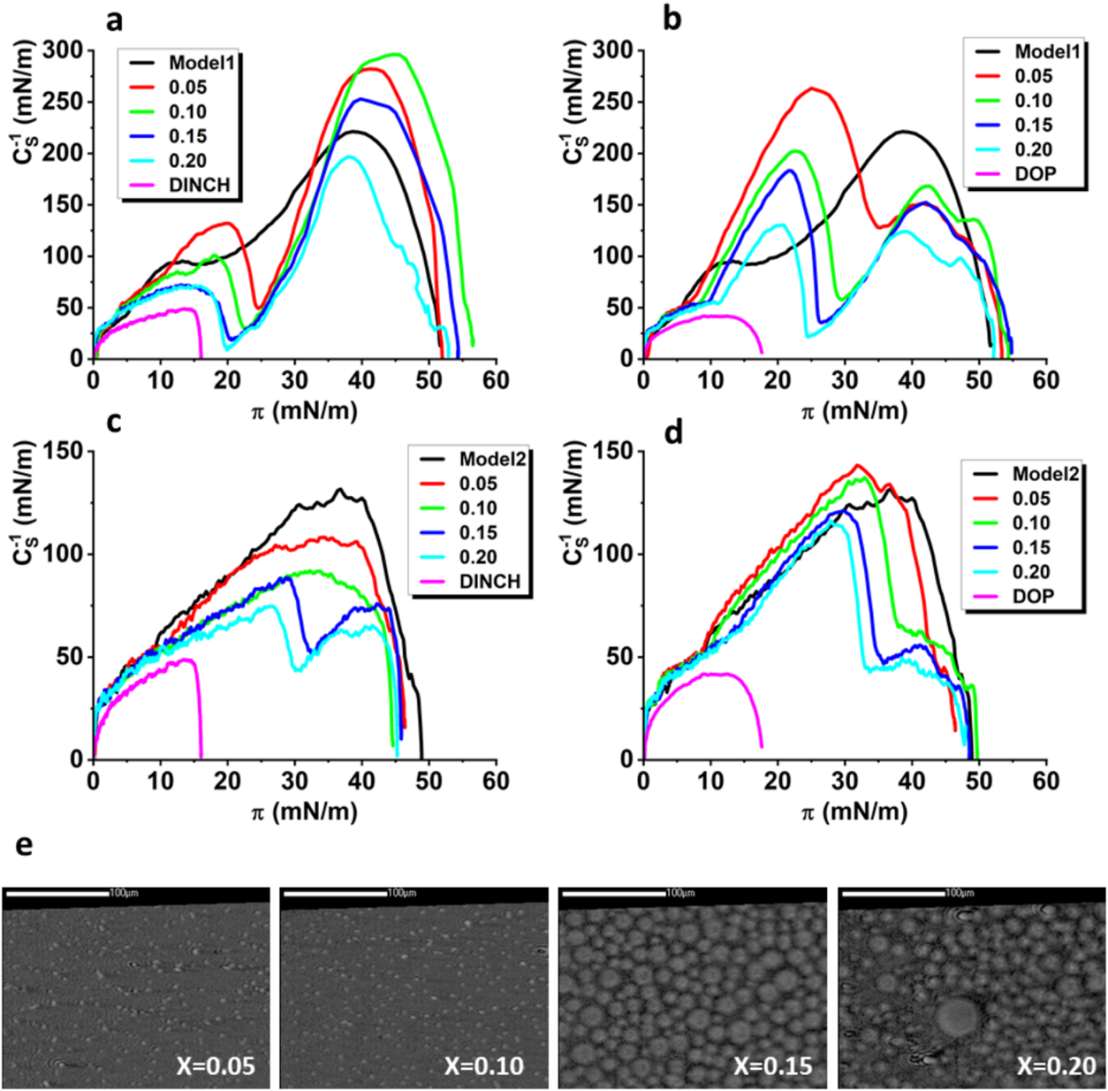
*C*_S_^–1^-π curves for the systems: (a) Mod1_DINCH, (b) Mod1_DOP, (c) Mod2_DINCH, (d) Mod2_DOP, (e) BAM images recorded at 15 mN/m for the system Mod1_DINCH. Mole ratios of DINCH are indicated in the images. The scale bar indicates 100 μm.

At first glance, DINCH affects the *C*_S_^–1^-π curves similarly to DEHP. The introduction of DINCH into the model Mod1 membrane, already at X­(DINCH) = 0.05, causes the appearance of a deep minimum in the *C*_S_^–1^-π curve. The location of this minimum correlates with the pressure at which the first multilayer aggregates are formed (Figure S8B). At higher π values above this minimum, a significant increase in the *C*_S_^–1^ value can be observed. The condensing effect exerted by DINCH on the Mod1 monolayer is stronger than in the case of DEHP.

Additionally, before the minimum in the *C*_S_^–1^-π curve for X­(DINCH) = 0.05, a clear condensation effect is visible—the *C*_S_^–1^ values are higher than for the Mod1 monolayer. For X­(DINCH) = 0.10, the *C*_S_^–1^-π curve overlaps with that measured for the undoped monolayer, while for larger X­(DINCH), a slight expansion effect can be observed.

A small addition of DOP (X­(DOP) = 0.05) to the Mod1 monolayer also causes an increase in its condensation. However, for this system, the curve is different. No minimum is observed around 20–25 mN/m. The compression modulus first reaches its maximum value, and a minimum in its course appears only later at π around 35 mN/m. This minimum, similarly to the previously discussed cases, corresponds to the surface pressure at which multilayer aggregates begin to nucleate. For the remaining X­(DOP) cases, the situation is somewhat more complicated. If the criterion of the highest *C*_S_^–1^ value were taken into account, in these cases, it is significantly lower than for the undoped monolayer, meaning that the addition of DOP leads to monolayer expansion. However, before the minimum on the *C*_S_^–1^-π curve, the situation is completely different—there, a sharp increase in the *C*_S_^–1^ value is observed. Therefore, it can be concluded that the addition of DOP causes strong condensation of the monolayer. However, it must be taken into account that the compression modulus is a global quantity determined for the monolayer as a whole. Multilayer aggregates appearing during compression disrupt the integrity of the monolayer; therefore, after the minimum on the *C*_S_^–1^-π curve, the compression modulus values for the DOP-enriched monolayer are significantly lower than for the initial Mod1 monolayer.

The question is how the conclusions drawn from the *C*_S_^–1^-π curves translate into the morphology changes observed using BAM microscopy. Figure 4e shows BAM images taken for the Mod1_DINCH system at π = 15 mN/m. Here, the conclusions drawn from the analysis of the *C*_S_^–1^-π curves fully coincide with the observed morphology. For X­(DINCH) = 0.05 and 0.10, progressive condensation was inferred, and this is indeed the case—the BAM images show the presence of numerous small condensed domains. In turn, for X­(DINCH) = 0.15 and 0.20, a slight expansion was inferred, and the BAM images also confirm this—the area of the monolayer in the LE state increases. As for the entire set of BAM images for the Mod1_DINCH system (Figure S8A), the presence of DINCH in the monolayer does not drastically affect its morphology. For lower DINCH contents, a decrease in the diameters of the condensed domains and their density is observed compared to the undoped monolayer, while for X­(DINCH) = 0.15 and 0.20, the BAM images are similar to those recorded for the Mod1 monolayer.

Regarding the Mod1_DOP system, the full set of BAM images is provided in Figure S9A in the Supporting Information. The addition of DOP drastically changes the morphology of the Mod1 membrane. At π = 15 and 20 mN/m, regardless of X­(DINCH), the BAM images are completely homogeneous, i.e., there is complete fusion of the condensed domains associated with the strong condensation effect caused by this plasticizer.

For the Mod2_DINCH system, it can be observed that with increasing X­(DINCH), the *C*_S_^–1^ values gradually decrease, which means that DINCH exerts an expanding effect on this membrane model. At X­(DINCH) = 0.05 and 0.10, there is no minimum in the *C*_S_^–1^-π curve, which means that it can be assumed that DINCH molecules remain in the lipid matrix until the collapse pressure is reached. However, for X­(DINCH) = 0.15 and 0.20, a minimum appears in the *C*_S_^–1^-π curve at π around 35 mN/m, which is associated with the release of DINCH from these monolayers and the formation of multilayer aggregates. The effect of DOP on the packing of lipid molecules in the Mod2 monolayer is negligible. Up to surface pressures of around 30 mN/m, the *C*_S_^–1^-π curves overlap within the limits of measurement error. Again, at X­(DOP) = 0.05 and 0.10, no minimum occurs in the *C*_S_^–1^-π curve, while for X­(DOP) = 0.15 and 0.20, at π around 35 mN/m, a shallow minimum appears, associated with the separation of multilayer aggregates from the monolayer. Moreover, as can be seen in Figure S9B, the first multilayer aggregates nucleate already at lower π values, while their number increases rapidly after exceeding the π value corresponding to the minimum on the *C*_S_^–1^-π curve.

For these systems, the excess free energies of mixing were also calculated and presented in the form of Δ*G* ^exc^–*X*(plasticizer) graphs in Figure 5.

**Figure 5:**
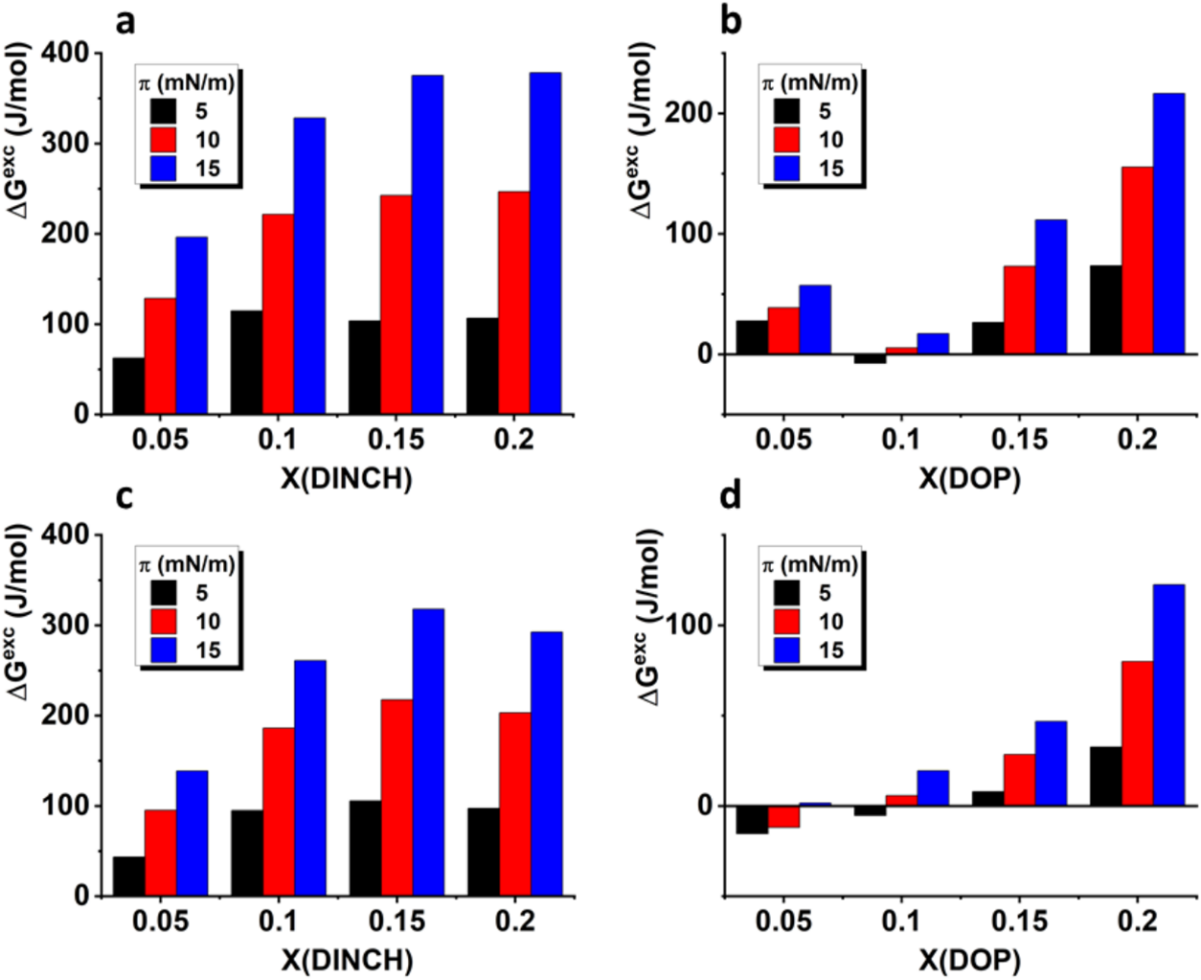
Δ*G* ^exc^–*X*(phthalate) plots for the studied systems: (a) Mod1_DINCH, (b) Mod1_DOP, (c) Mod2_DINCH, (d) Mod2_DOP.

For the Mod1_DINCH system, the sign of the Δ*G* ^exc^ is positive, and its values are clearly higher than for the systems discussed so far—for π = 15 mN/m they reach about 400 J/mol. Therefore, it can be concluded that the interactions of DINCH with the lipids of the model membrane are energetically unfavorable. The strong increase in Δ*G* ^exc^ with increasing π correlates with the release of DINCH molecules from the lipid monolayer and the nucleation of multilayer aggregates even at relatively low surface pressure values below 20 mN/m (see Figure S8A,B). In the Mod1_DOP system, the sign of the Δ*G* ^exc^ is also positive, but the values are much smaller than in the Mod1_DINCH system. For *X*(DOP) = 0.05, the Δ*G* ^exc^ values are very small, and for *X*(DOP) = 0.10, they oscillate around 0 J/mol. It can therefore be concluded that with a small addition of DOP, the miscibility of this substance with the lipids of the monolayer is ideal. For *X*(DOP) = 0.15 and 0.20, the Δ*G* ^exc^ values are slightly higher, reaching about 200 J/mol for *X*(DOP) = 0.20 and π = 15 mN/m. It can therefore be concluded that a greater addition of DOP disrupts the interactions between lipid molecules in the Mod1 monolayer, which become energetically unfavorable.

The Δ*G* ^exc^–X­(DINCH) dependence for the Mod2_DINCH system is almost identical to that for the Mod1_DINCH system discussed above. Δ*G* ^exc^ has a positive sign, and its values are similar to those in the mentioned system. This means that the interactions between DINCH molecules and Mod2 membrane lipids are energetically unfavorable. The Mod2_DOP system also resembles the Mod1_DOP system described above. For *X*(DOP) = 0.05 and 0.10, the Δ*G* ^exc^ values oscillate around 0 J/mol, and for *X*(DOP) = 0.15, they are positive but very small. Only for *X*(DOP) = 0.20 are the Δ*G* ^exc^ values slightly larger, but the largest observed value in this system is only about 100 J/mol, so it can be concluded that the miscibility of DOP with the lipids of the Mod2 monolayer is ideal.

### The Effect of Longer-Chain Phthalates: DIDP, DDP, and DTP, on Apical Membrane Models

Long-chain phthalates, such as diisodecyl phthalate, di-*n*-decyl phthalate, or di-*n*-tridecyl phthalate, are also currently used as plasticizers for vinyl polymers.^84,85^ Therefore, in our studies, we investigated the effects of these three plasticizers on model apical membranes. π-*A* isotherms for these systems are presented in Figure S10 in the Supporting Information, while the *C*_S_^–1^-π plots are shown in Figure 6.

**Figure 6:**
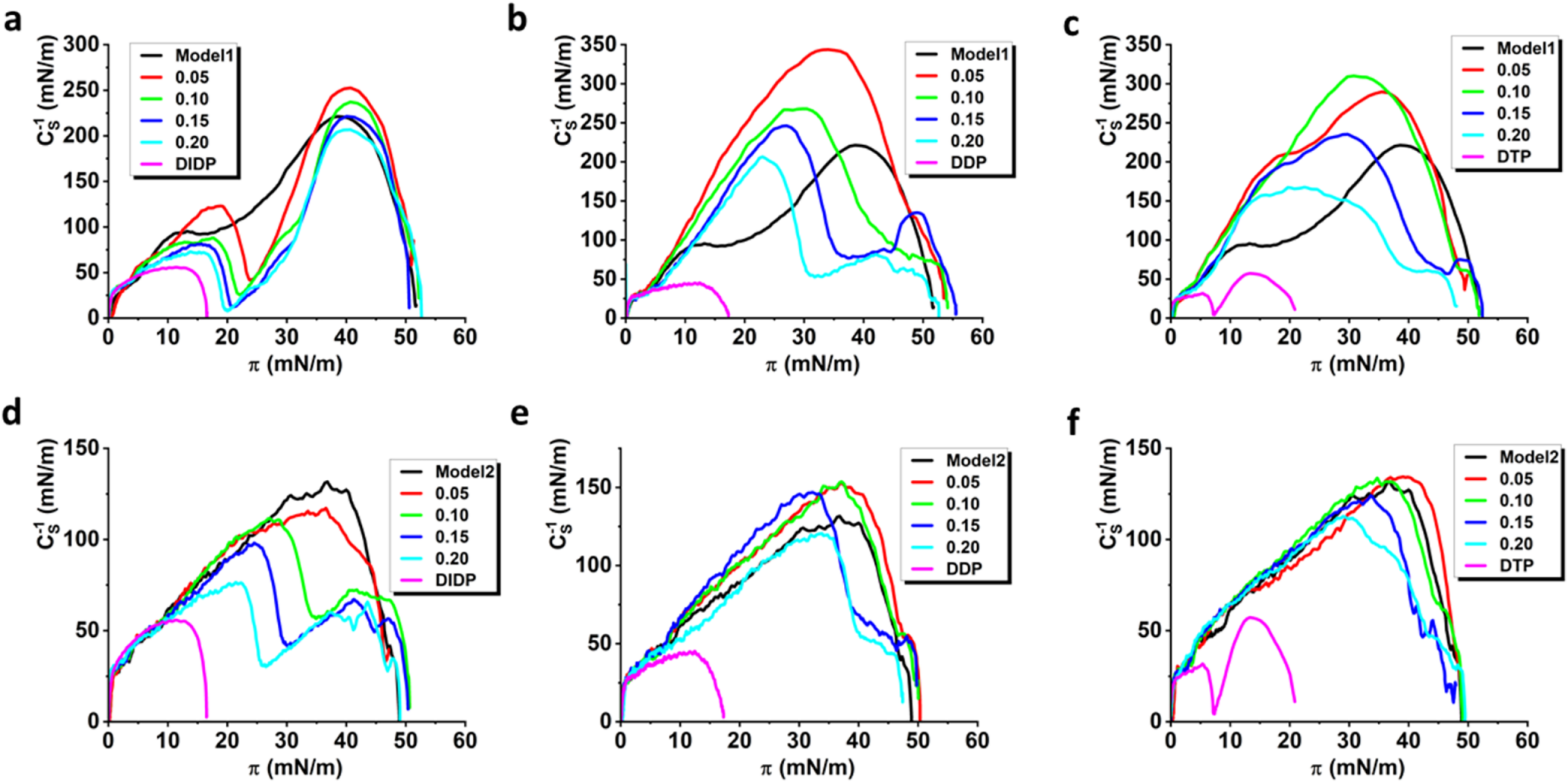
*C*_S_^–1^-π curves for the investigated systems: (a) Mod1_DIDP, (b) Mod1_DDP, (c) Mod1_DTP, (d) Mod2_DIDP, (e) Mod2_DDP, (f) Mod2_DTP.

DIDP introduced into the Mod1 monolayer behaves similarly to DEHP and DINCH. First, a deep minimum appears in the *C*_S_^–1^-π curve, the location of which correlates with the surface pressure at which multilayer aggregates begin to appear on the monolayer surface (see Figure S11B). Before this minimum, for *X*(DIDP) = 0.05, a condensing effect is observed—*C*_S_^–1^ values are higher than for the undoped monolayer. For *X*(DIDP) = 0.10, the *C*_S_^–1^-π curve up to π about 20 mN/m overlaps with the curve measured for Mod1, while for larger X­(DIDP), the *C*_S_^–1^ values in this region are lower than for Mod1, indicating a slight reduction in monolayer condensation. The maximum *C*_S_^–1^ values at X­(DIDP) = 0.05 and 0.10 are slightly higher than for the Mod1 membrane, indicating a slight condensing effect and confirming that some DIDP molecules remain in the lipid matrix until the monolayer collapse pressure. The full set of BAM images for the Mod1_DIDP system is shown in Figure S11A in the Supporting Information. For *X*(DIDP) = 0.05 and 0.10, the BAM images look very similar to those for the initial Mod1 monolayer, although at π = 20 mN/m the monolayer is almost homogeneous, confirming the condensing effect exerted by DIDP on the model membrane. For *X*(DIDP) = 0.15 and 0.20 at π = 5 and 10 mN/m, the BAM images are similar to those recorded for the Mod1 monolayer, while at π = 15, the BAM images clearly differ. In the presence of DIDP, progressive fusion of the condensed domains is visible, although an increased proportion of the monolayer in the LE state is also visible, which would confirm the small expansion effect postulated for DIDP at these values of *X* and π.

The introduction of DDP leads to a significant increase in monolayer condensation, and the compression modulus reaches a maximum value of c.a. 350 mN/m (an increase of over 100 mN/m). The maximum of the *C*_S_^–1^-π curve shifts with increasing *X*(DDP) toward lower values of π, and up to *X*(DDP) = 0.15 is at a higher level than for Mod1. At *X*(DDP) = 0.15 and 0.20 in the course of the *C*_S_^–1^-π curve at π around 35 mN/m, a minimum appears associated with the separation of phthalate from the monolayer and the formation of multilayer aggregates. The BAM images collected in Figure S12B prove that the nucleation of the first multilayer aggregates occurs at lower surface pressure values than could be inferred from the course of the *C*_S_^–1^-π curve. The full set of BAM images for the Mod1_DIDP system is presented in Figure S12A. The addition of DDP to the Mod1 monolayer causes the fusion of condensed domains and, consequently, homogenization of the monolayer. At *X*(DDP) = 0.05, 0.10, and 0.15, the monolayer is completely homogeneous; only at π = 5 mN/m are remnants of the boundaries between some domains visible—remnants of the LE phase. Only an increase in *X*(DDP) to 0.2 causes the reappearance of condensed domains separated by regions in the LE state, and the monolayer does not achieve full homogeneity.

The addition of DTP also causes a profound increase in monolayer condensation, manifested by a significant increase in *C*_S_^–1^. The maximum *C*_S_^–1^ values for *X*(DTP) = 0.05 and 0.10 again approach 350 mN/m and are over 100 mN/m higher than those observed for the undoped membrane. The condensing effect is also noticeable for *X*(DTP) = 0.15 and 0.20, although for *X* = 0.20, the maximum *C*_S_^–1^ value is lower than for the Mod1 monolayer. For all tested *X*(DTP), there is no minimum in the *C*_S_^–1^-π curve, i.e., until the monolayer collapse, there is no rapid DTP release from the model membrane, although in the BAM images (Figure S13B), the appearance of a few small 3D aggregates can be observed at π at the level of 25–30 mN/m.

The complete set of BAM images for the Mod1_DTP system is presented in Figure S13A in the Supporting Information. In general, similar to DDP, the addition of DTP also causes fusion of condensed domains and homogenization of the monolayer. However, as discussed previously, the texture of the Langmuir monolayer need not be uniform throughout its entire extent, hence some BAM images show that homogeneous regions of the LC monolayer may be in equilibrium with regions where separate condensed domains and remnants of the LE phase are observed.

The strong condensation effects described above are not observed for model 2, which imitates the nonraft regions of the apical membrane. The addition of DIDP to the Mod2 monolayer at X­(DIDP) = 0.05 has practically no effect on its condensation, and the *C*_S_^–1^-π curve coincides with that measured for the Mod2 monolayer. Increasing *X*(DIDP) causes an expansion of the monolayer, manifested in a successive decrease in *C*_S_^–1^ values. Additionally, from *X*(DIDP) = 0.1, a minimum appears in the *C*_S_^–1^-π curve associated with the nucleation of DIDP aggregates from the lipid monolayer. Regarding DDP and DTP, the addition of these phthalates within the mole fraction range of 0.05 to 0.20 does not affect monolayer condensation. The *C*_S_^–1^-π curves overlap within the experimental error. It is worth emphasizing that the addition of DDP or DTP, regardless of the mole fraction of these substances, does not cause a minimum in the *C*_S_^–1^-π curve. Therefore, it can be concluded that these phthalate molecules remain incorporated into the lipid matrix until the monolayer collapse pressure is reached.

Excess free energies of mixing, Δ*G* ^exc^, were calculated for the discussed systems. Δ*G* ^exc^–*X*(phthalate) plots are presented in Figure 7.

**Figure 7:**
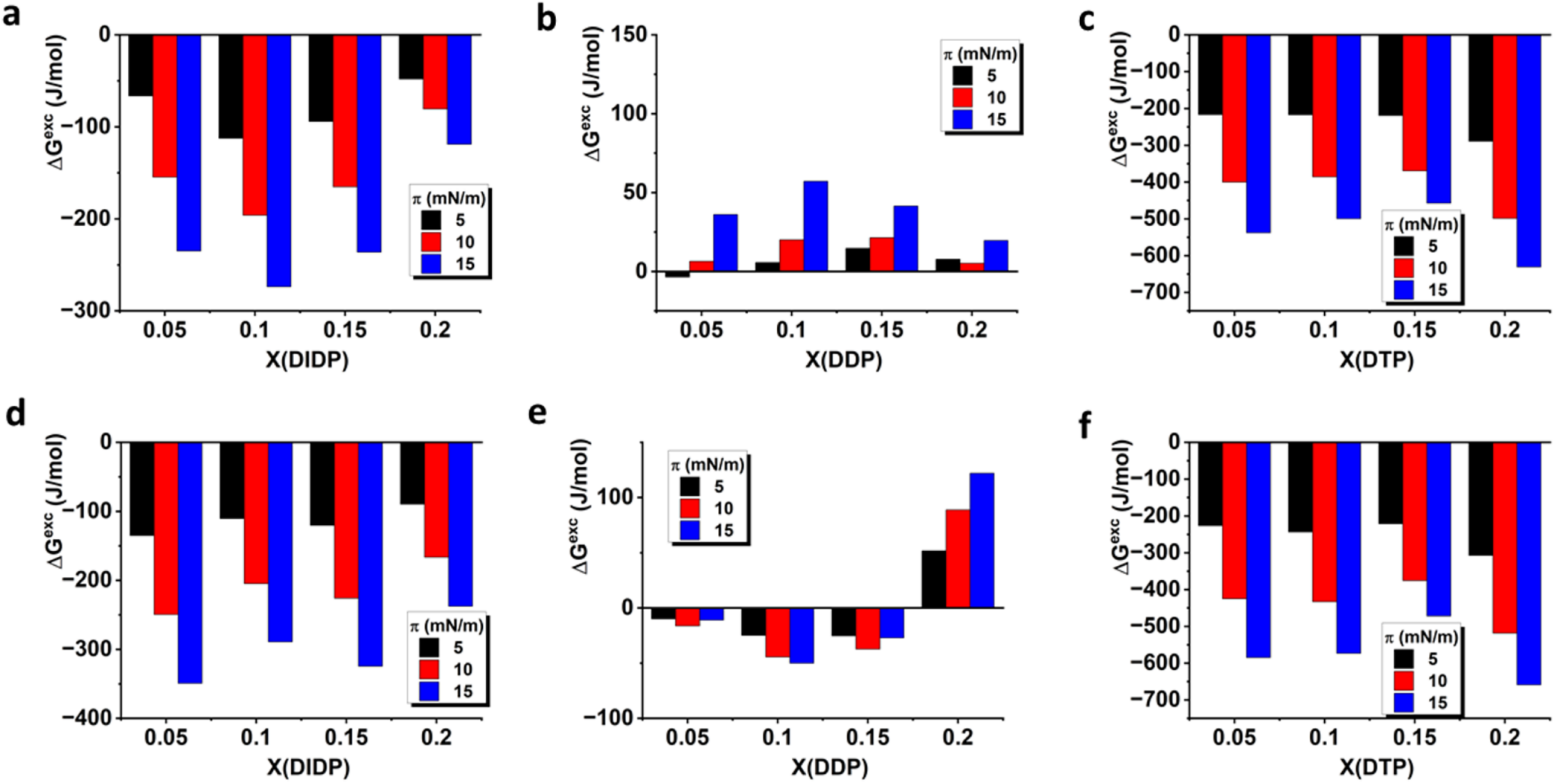
Δ*G* ^exc^–*X*(phthalate) plots for the systems: (a) Mod1_DIDP, (b) Mod1_DDP, (c) Mod1_DTP, (d) Mod2_DIDP, (e) Mod2_DDP, (f) Mod2_DTP.

For the phthalates discussed in this chapter, the Δ*G* ^exc^–*X*(phthalate) dependencies are very similar for both models, so they will be discussed simultaneously. For the Mod1_DIDP and Mod2_DIDP systems, Δ*G* ^exc^ has a negative sign, and its values at π = 15 mN/m reach approximately −300 J/mol. Therefore, it can be concluded that the interactions between lipid molecules of both model systems and DIDP molecules are energetically favorable. In the Mod1_DDP and Mod2_DDP systems, Δ*G* ^exc^ values oscillate around 0 J/mol. In the Mod1-DDP system, the sign of Δ*G* ^exc^ is positive, and in the Mod2_DDP system, negative. However, since the values are very small, in the range of −50 to 50 J/mol, it can be concluded that miscibility in these systems is close to ideal. As for the Mod1_DTP and Mod2_DTP systems, the sign of Δ*G* ^exc^ is negative, and its values are significant, reaching around −600 J/mol at π = 15 mN/m. Therefore, it can be concluded that the interactions between the lipid molecules of both models and the DTP molecules are energetically favorable, and the DTP molecules bind strongly to the lipid matrix of the model membranes.

### The Structure of the Phthalate Molecule vs Its Influence on the Physical Properties of Model Apical Membranes

Lipid rafts are characterized by significant stiffness, and the packing of lipid molecules within them can be periodic.^76,86^ Therefore, it was interesting to investigate the effect of phthalate incorporation on the formation of 2D crystalline nanodomains in the studied monolayers. All these studies were performed at π = 15 mN/m and for the largest *X*(phthalate) of 0.2. The results of the GIXD studies are presented in Figure 8.

**Figure 8:**
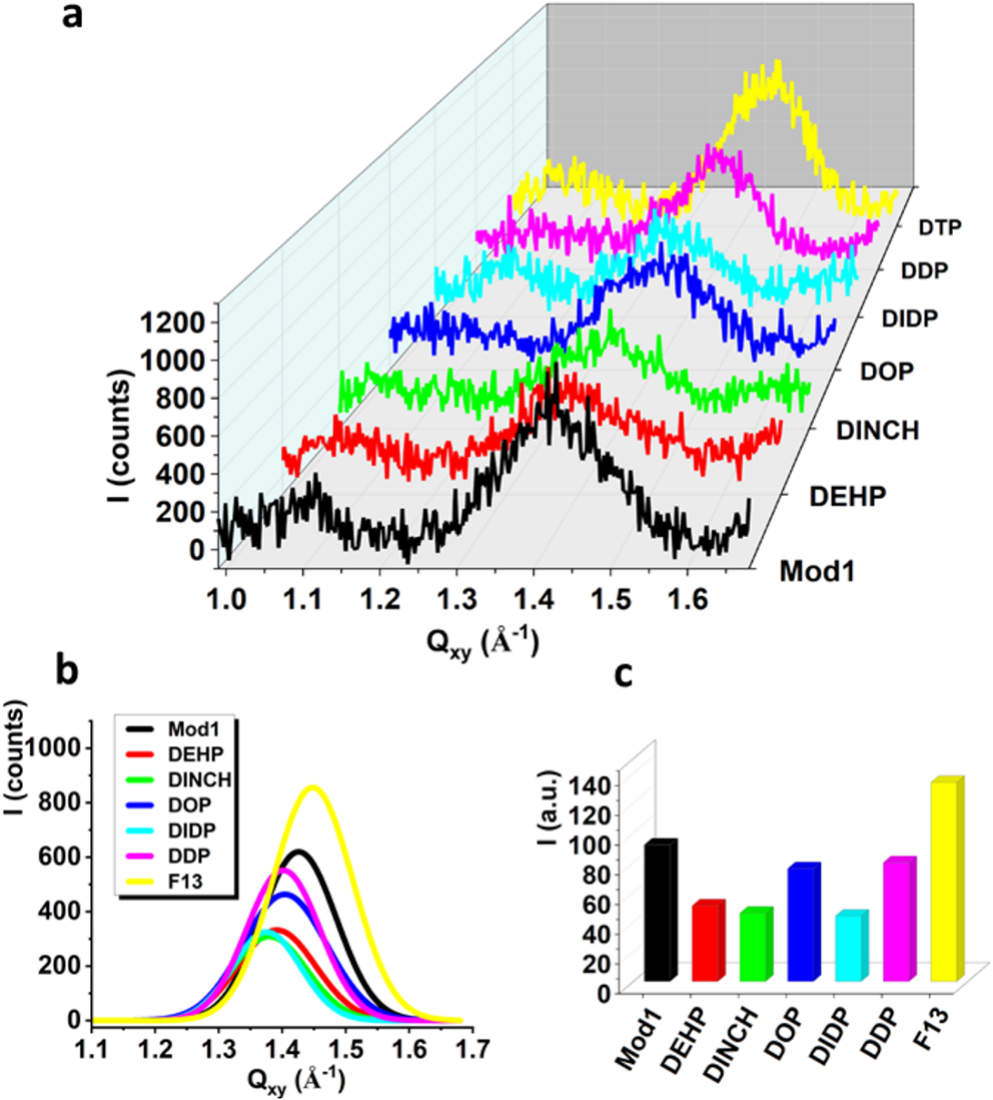
(a) *I*(*Q*_*xy*_) Bragg peak plots for the investigated monolayers. (b) Gauss curve fits of the Bragg peaks, (c) comparison of the Bragg peak intensities.

A weak diffraction signal was observed for all phthalate-enriched Mod1 monolayers. The addition of phthalate does not quench the diffraction signal, meaning that 2D crystalline nanodomains still exist in the phthalate-enriched monolayers. The intensity of the diffraction signal can be correlated with the number of nanodomains coherently scattering the synchrotron beam.^65,66^ Branched-chain plasticizers, DEHP, DINCH, and DIDP, reduce the signal intensity by approximately half, indicating that the number of scatterers at the water/air interfaces significantly decreases in their presence. The phospholipids comprising model 1, DPPC, and egg-SM, contain unbranched palmitic chains. The presence of branched chains nearby hinders the periodic packing of lipids in the monolayer plane, resulting in a smaller number of nanodomains. DOP and DDP slightly reduce the intensity of the diffraction signal, while the incorporation of DTP molecules into the monolayer increases the signal intensity by approximately 40%. The presence of phthalates in the monolayer does not affect the half-width of the Bragg peaks. The half-width of the Bragg peaks is inversely proportional to the extent of 2D crystallinity, or, to simplify, the diameter of the nanodomains.^65,66^ Thus, the addition of phthalates does not affect the average diameter of 2D crystalline nanodomains. As can be seen in Figure 8b, the addition of phthalate affects the position of the maximum in the *I*(*Q*_*xy*_) plot. Only the addition of DTP shifts this maximum toward higher *Q*_*xy*_ values, which is a manifestation of monolayer condensation at the molecular level. The remaining phthalates shift the Bragg peak maximum toward lower *Q*_*xy*_ values. For DDP and DOP, these shifts are slight, but for branched-chain phthalates, they are more pronounced. Such shifts indicate an increase in the unit cell area, i.e., monolayer expansion at the molecular level.

Our studies were intended to answer the four questions posed in the introduction:

(1) Do phthalates incorporate into the model membranes? (2) Are there noticeable differences in the interactions of phthalates with models of raft and nonraft regions of the apical membrane? (3) Whether and to what extent do they influence their physical properties and morphology? (4) Does the effect exerted by phthalates depend on the structure of the aliphatic chains of the phthalate molecule?

Regarding the first question, the answer is yes, they do build in. However, there are a few nuances to consider. In the literature on the use of Langmuir monolayers as models of biological membranes, it is often assumed that the arrangement of hydrocarbon chains in a monolayer resembles the arrangement of these chains in a bilayer (e.g., in liposomes or a real cell membrane) when the Langmuir monolayer is compressed to approximately 30 mN/m.^87,88^ Phthalates exhibit surface activity and form Langmuir monolayers, but these monolayers are characterized by limited stability and collapse at surface pressures of the order of 15–20 mN/m.^20^ When a substance of this type, or a xenobiotic without any surface activity, is incorporated into a lipid Langmuir monolayer, it can be expected that during compression, after exceeding a certain threshold value of surface pressure, the xenobiotic molecules will separate from the monolayer, forming multilayer aggregates on its surface. The question arises: what is this threshold surface pressure in a given case, and whether it is significantly lower than 30 mN/m? If it is significantly lower, one could argue that the xenobiotic in question would not incorporate into a real membrane at all. In our study, we have two models of raft (Mod1) and nonraft (Mod2) regions of the apical membrane. Up to a pressure of 20 mN/m, which is 5 mN/m higher than the collapse pressure of phthalate monolayers, we do not observe multilayer aggregates. Phthalates are incorporated into both models. For Mod2, the surface pressures at which multilayer aggregates start to nucleate are high, on average exceeding 35 mN/m, so there is no significant problem here—phthalates are incorporated into the model under physiological conditions. The situation is different for Mod1—in this case, numerous multilayer aggregates form in the surface pressure range of 20–30 mN/m (depending on the phthalate used). However, not all phthalate molecules are released. Significant increases in the compressibility modulus at high (>30 mN/m) π, or significant changes in the monolayer morphology, indicate that some phthalate molecules remain in the Mod1 membrane until it collapses. The issues discussed here are also related to the problem of selecting concentrations, or rather molar proportions, of phthalates for our studies. We used molar proportions of phthalates: 0.05, 0.10, 0.15, and 0.20. Of course, as always in modeling studies, these proportions are very large compared to those expected under real-world conditions. However, each instrumental method has its limitations, and we select a set of concentrations to provide a chance to observe differences and to observe the effect of concentration on the phenomena being studied. In our studies, it is worth emphasizing the particularly significant effect on Mod1 membranes at the lowest *X*(phthalate) = 0.05—typically, the largest increases in compressibility modulus were observed for this molar fraction. On the other hand, larger molar proportions of xenobiotics allow discussion of the potential accumulation of these substances in model membranes, or, in the case of a series of xenobiotics, their ranking by affinity for lipid structures.

The next question was whether there are significant differences in the interaction of phthalates with models of raft and nonraft regions of apical membranes. The answer is yes, there are enormous differences in these interactions. Phthalates exert a very large influence on the raft region model (Mod1) and only a small influence on the physical properties of the nonraft region model (Mod2). This raises a more fundamental issue concerning the modeling of biological membranes in general. The appropriate selection of the lipid composition of such a membrane is crucial.^50^ Based on the available scientific literature, we attempted to recreate the composition of apical membranes as faithfully as possible. We also considered the limitations of our model—as the name suggests, it is a monolayer model, meaning we are not modeling the entire membrane (bilayer), but only one of its lamellae. Naturally, if we want to model the part of the membrane that is in contact with the outside world, it must be the outer lamella. This membrane contains raft regions crucial for its function, but also nonraft regions of lower condensation. Therefore, to model the apical membrane, we proposed two different models. This approach proved very fruitful and allowed us to demonstrate significant differences. Phthalates can be incorporated even in large amounts (*X*(phthalate) = 0.20) into the model nonraft membrane (Mod2) without significantly affecting the physical properties of such a membrane. The situation is completely different in the lipid raft model (Mod1), where even small additions of phthalate can lead to significant changes in the mechanical properties and morphology of the membrane. Considering that lipid rafts play a key role in the functioning of the apical membrane, this is a very important conclusion. Phthalates can incorporate into lipid rafts, disrupting normal interactions between lipids in these domains and ultimately leading to their dysfunction. This suggests a mechanism of phthalate toxicity at the membrane level. Our results indicate that phthalates can perturb lipid packing and lateral organization in the raft-like monolayer model, which may impair membrane-associated processes in apical membranes. However, because the present study uses Langmuir monolayers, these findings should be interpreted as a physicochemical mechanism at the model membrane level and require validation in bilayer and cell-based systems.

The answer to the third and fourth questions regarding the effect of phthalates on the physical properties of the model apical membrane depends directly on the structure of the phthalate being studied. Of course, when we refer to the apical membrane here, we are referring to the model of the raft regions of this membrane, because, as discussed in the previous section, the effect of phthalates on the model of nonraft regions is weak and poorly correlated with the phthalate structure. A sound discussion should focus on “membrane-level toxicology”, meaning it should identify which phthalates are more destructive to membranes and which are less so, and how this relates to their structure. Discussing this issue requires developing comparative criteria based on the experimental techniques used. In our studies, we discussed the effect of phthalates on the mechanical properties of model membranes (π-A isotherms, *C*_S_^–1^-π curves), intermolecular interactions within monolayers (Δ*G* ^exc^–*X*(phthalate) curves), monolayer morphology (BAM microscopy), and two-dimensional crystallinity within model lipid rafts (GIXD technique). So when does a given phthalate have an exceptionally negative effect on the membrane, and when is it relatively neutral? It can be assumed that if the incorporation of a given phthalate does not lead to drastic changes in the membrane’s mechanical properties and does not significantly affect the membrane’s morphology, the phthalate can be considered neutral. However, the greater the observed impact, the more harmful the phthalate is. Regarding the Δ*G* ^exc^ criterion, excessively strong interactions of phthalate with monolayer lipids, manifested by significant negative values of this function, are not beneficial. This may indicate significant accumulation of phthalates in the membrane, and the strong interaction between phthalate and lipid molecules can lead to significant changes in its mechanical properties and morphology. Conversely, excessively positive Δ*G* ^exc^ values are also unfavorable, as they can indicate serious disruptions in the interactions between lipid molecules in the model membrane caused by the presence of xenobiotic molecules. Therefore, for phthalate, which we can consider neutral for the membrane, the most favorable situation is when Δ*G* ^exc^ values are close to 0 J/mol. Regarding two-dimensional crystallinity, it would be best if phthalate did not affect the number of 2D crystalline domains or their structure.

The introduction of the above criteria enables the ranking of phthalates in terms of their membrane activity. One of the goals was to compare the effect of DEHP (an old, traditional plasticizer) with its substitutes. From the perspective of the above criteria, DEHP behaves quite favorably—it affects the elasticity of the Mod1 monolayer only moderately and weakly influences the membrane morphology. Δ*G* ^exc^ for smaller *X*(DEHP) is close to 0 J/mol. In contrast, DEHP significantly reduces the number of crystalline nanodomains. Two other plasticizers that moderately affect the elasticity of the Mod1 monolayer and its morphology are DINCH and DIDP. For DINCH, Δ*G* ^exc^ values are positive and slightly too high, reaching up to 400 J/mol at π = 15 mN/m, which may lead to the conclusion that the presence of DINCH significantly disrupts the lipid packing of the Mod1 monolayer. For DIDP, the Δ*G* ^exc^ sign is negative, but the absolute ΔG^exc^ values are smaller than for DINCH—approximately 250 J/mol at π = 15 mN/m. Both of these plasticizers, similarly to DEHP, reduce the number of 2D crystalline nanodomains within the model lipid rafts. In turn, the addition of unbranched-chain phthalates, i.e., DOP, DDP, and DTP, leads to significant changes in the properties of the model Mod1 membrane. These phthalates primarily cause a very strong condensing effect, manifested by a significant increase in the *C*_S_^–1^ values. This significant stiffening of the model lipid raft is a disadvantage. It is important to remember that real lipid rafts are anchoring platforms for numerous transmembrane proteins, and appropriate interactions of the raft lipids with the protein impart the desired conformation and enable the protein to function. A significant change in lipid packing within the raft can deactivate specific proteins or prevent the formation of protein complexes. The significant increase in condensation of the model membrane after the addition of unbranched-chain phthalates also manifests itself in a drastic change in membrane morphology—the presence of these phthalates causes fusion of condensed domains and homogenization of the monolayer. Even assuming, as other authors have suggested, that lipid rafts aggregate in the apical membrane to form larger raft regions,^47^ these regions are still separated by nonraft regions,^48^ meaning that at the mesoscale visualized using BAM, a favorable situation is when we observe separate condensed domains and regions in the LE state, rather than a homogeneous monolayer in the LC or S state. Regarding the Δ*G* ^exc^ criterion, it differentiates phthalates with unbranched chains. For DOP and DDP, Δ*G* ^exc^ values are close to 0 J/mol— we interpret this situation as favorable. For DTP, however, Δ*G* ^exc^ has a negative sign, and the absolute values of this function are large, reaching up to 700 J/mol at π = 15 mN/m. This means that DTP molecules interact very strongly with membrane lipids, which in real-world conditions could lead to significant accumulation of this plasticizer in the apical membrane and, consequently, serious changes in its structure and function. DOP and DDP have a weak effect on the number of crystalline nanodomains, while the presence of DTP significantly increases their number, which, as discussed above, is not a favorable phenomenon.

To summarize this discussion, it can be concluded that all tested phthalates incorporate into the model lipid rafts of the apical membrane. Branched-chain phthalates have little effect on the physical properties and morphology of the model membrane, while incorporation of unbranched-chain phthalates leads to significant and unfavorable changes in the properties of the model system. The plasticizers DINCH and DIDP affect membrane properties similarly to DEHP, but toxicological studies indicate their much lower toxicity.^80,82^ Therefore, among the DEHP substitutes tested, DINCH and DIDP showed the weakest perturbation of the raft-like monolayer in our model system. These compounds should be considered promising candidates for further evaluation, whose superior toxicological safety should be confirmed *in vivo*.

## Conclusions

Current research indicates that the incorporation of xenobiotics into apical membranes and the disruption of lipid raft function may be a direct cause of numerous diseases and cancer. In our study, we used Langmuir lipid monolayers as models of both raft and nonraft regions of the apical epithelial membrane. DEHP, the most common plasticizer, and its current substitutes were incorporated into these models. The studies demonstrated that phthalates incorporate into both models of the apical membrane. However, their presence in the nonraft regions of the model does not lead to significant changes in its physicochemical properties. However, the presence of phthalates in the model raft membrane causes significant changes in its physical properties, adversely modifying its mechanical properties, domain morphology, and crystalline structure. *O*-phthalic acid diesters with long, unbranched alkyl chains disrupt the structure of the model rafts to a greater extent than DEHP, which they were intended to replace. However, the incorporation of phthalates with *iso*-branched chains does not cause such drastic changes in the physical properties of the model rafts. These compounds affect the model membrane similarly to DEHP. However, scientific literature indicates that they are much less toxic; therefore, from the perspective of membrane activity, using these phthalates as DEHP replacements is recommended.

## Supplementary Material



## Notes

Characterization of one-component lipid monolayers formed by the lipids used for the models construction (π–*A* isotherms, *C*_S_^–1^–π curves), Bragg peaks *I*(*Q*_*xy*_) for egg-SM, DPPC, and cholesterol. Characterization of phthalate monolayers (π–*A* isotherms, *C*_S_^–1^–π curves); characterization of the model membranes enriched in the studied phthalates: All π–*A* isotherms, complete BAM documentation (PDF)
