# Supplementary material for: Langmuir Monolayers as Effective Models of Apical Epithelial Cell Membranes: Studies on the Effects of Phthalate Incorporation
Source: Langmuir. 2026 Apr 28;42(18):13046–60. doi: 10.1021/acs.langmuir.6c01119 (PMC13308999; doi:10.1021/acs.langmuir.6c01119)
Supplement: Supplementary file 1 [file la6c01119_si_001.pdf]

Supplementary Materials

to the article

Langmuir monolayers as effective models of apical epithelial cell membranes. Study on the effects of phthalate incorporation

Marcin Broniatowski[1*] and Paweł Wydro[2]

[1] Department of Environmental Chemistry, Faculty of Chemistry, The Jagiellonian University in Kraków, ul. Gronostajowa 2, 30-387 Kraków, Poland

[2] Department of Physical Chemistry and Electrochemistry, Faculty of Chemistry, The Jagiellonian University in Kraków, ul. Gronostajowa 2, 30-387 Kraków, Poland

[*]corresponding author, broniato@chemia.uj.edu.pl

I. Characterization of single-component monolayers formed by lipids used to create apical membrane models

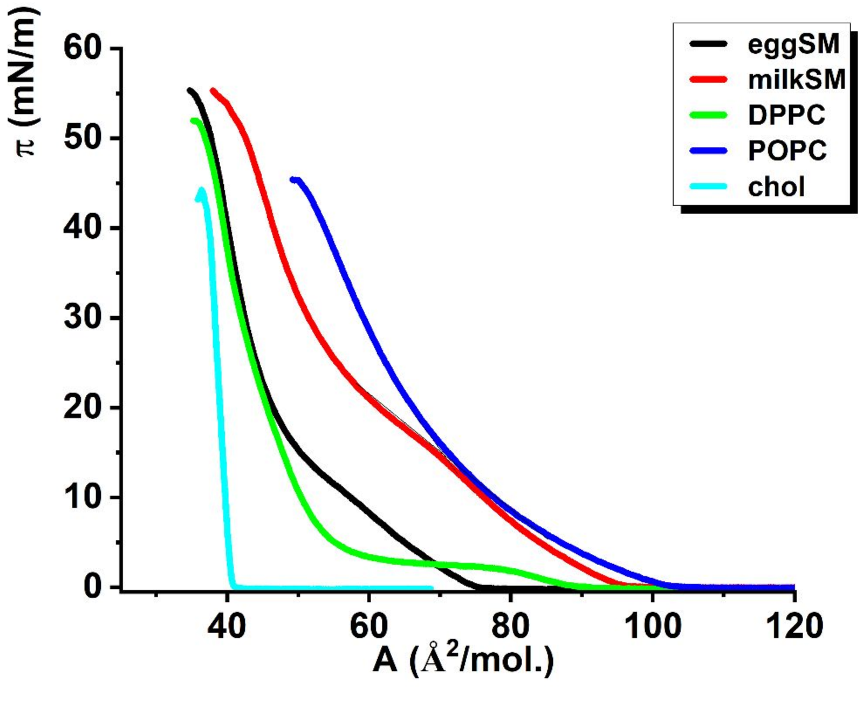

Fig. S1. π-A isotherms of single-component lipid monolayers.

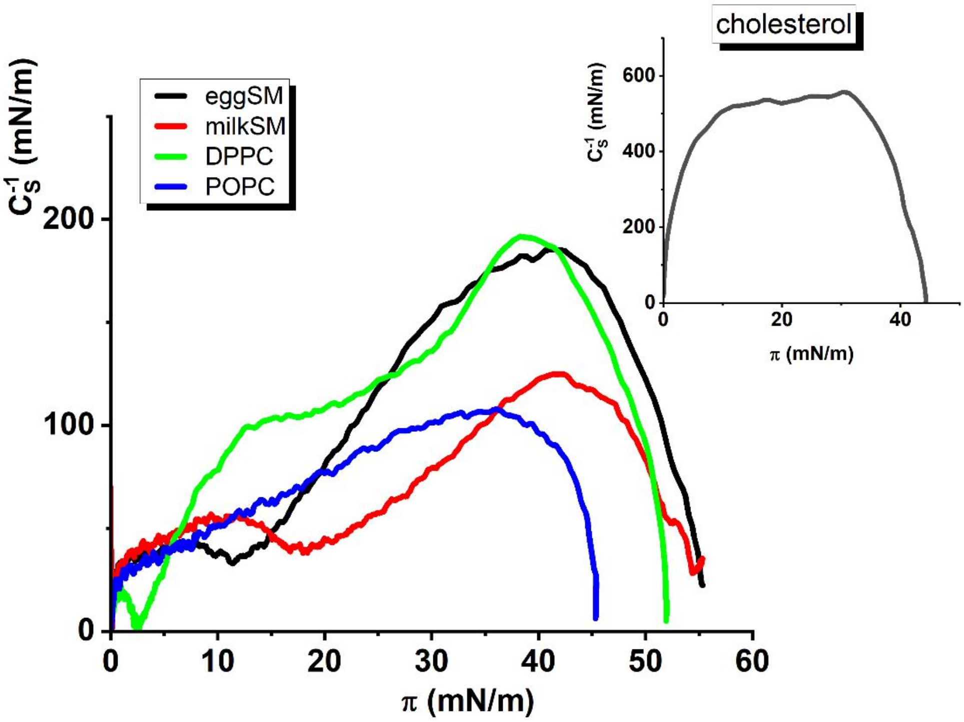

Fig. S2. C_S^{-1}-π curves of single-component lipid monolayers.

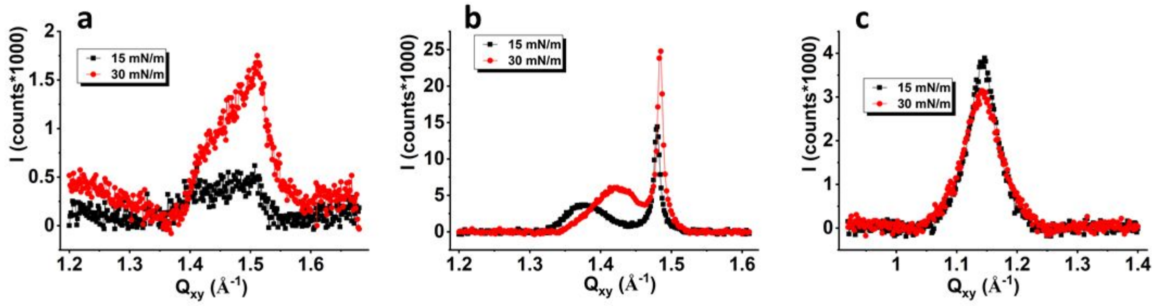

Fig. S3. Bragg peaks, I(Q_{xy}) for single-component lipid monolayers: a) eggSM, b) DPPC, c) cholesterol

II. Characterization of Langmuir monolayers formed by the studied phthalates

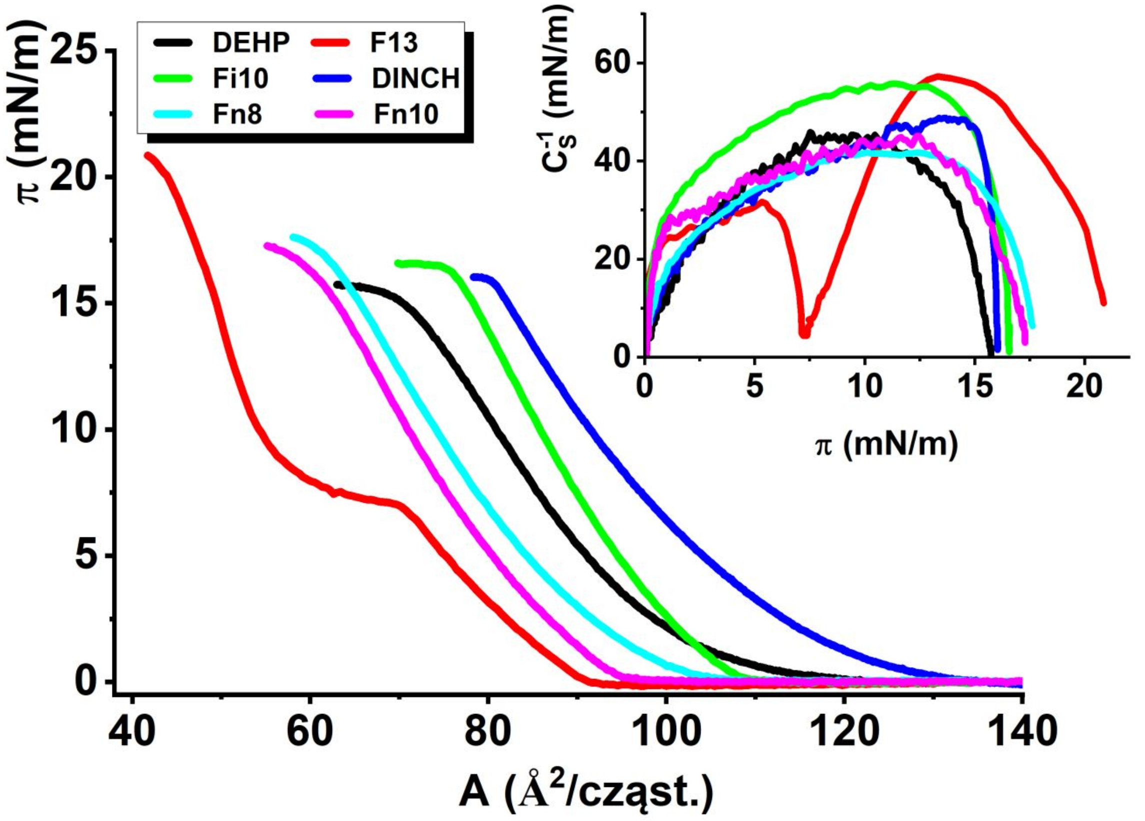

Fig. S4. π-A isotherms and C_S^{-1}-π curves of single-component monolayers formed by the studied plasticizers.

III. Characterization of the model membranes enriched in DEHP

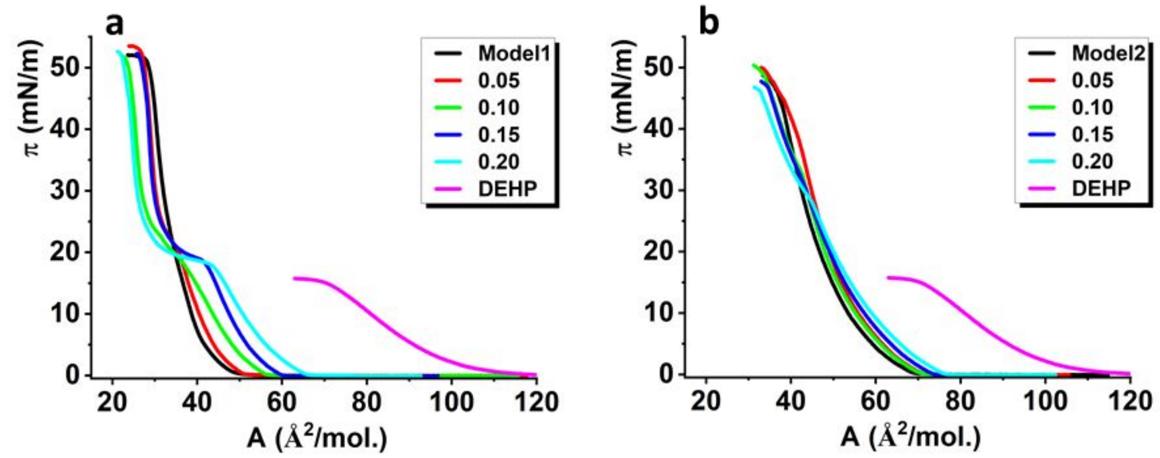

Fig.S5. π-A isotherms for the systems: a) Mod1_DEHP, b) Mod2_DEHP

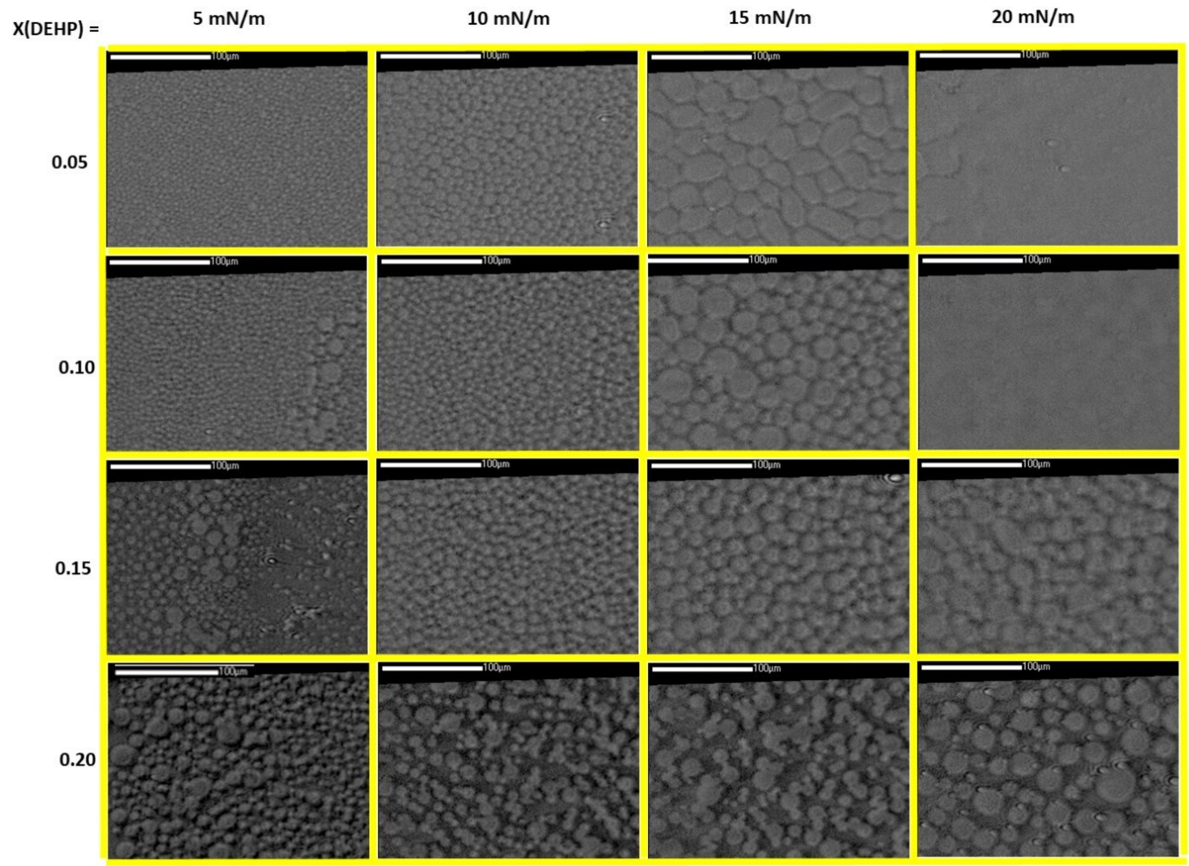

Fig. S6A. Selected BAM images for the system Mod1_DEHP.

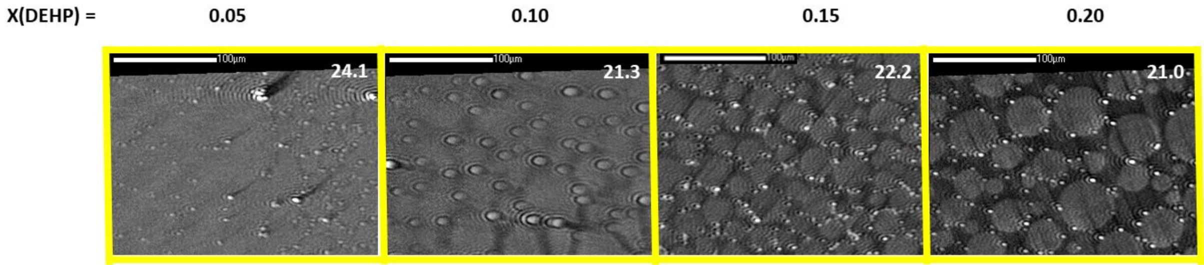

Fig. S6B. Aggregation in the system Mod1_DEHP. The numbers in the images indicate the surface pressure at which 3D aggregates appeared.

IV. Characterization of the model membranes enriched in DINCH and DOP

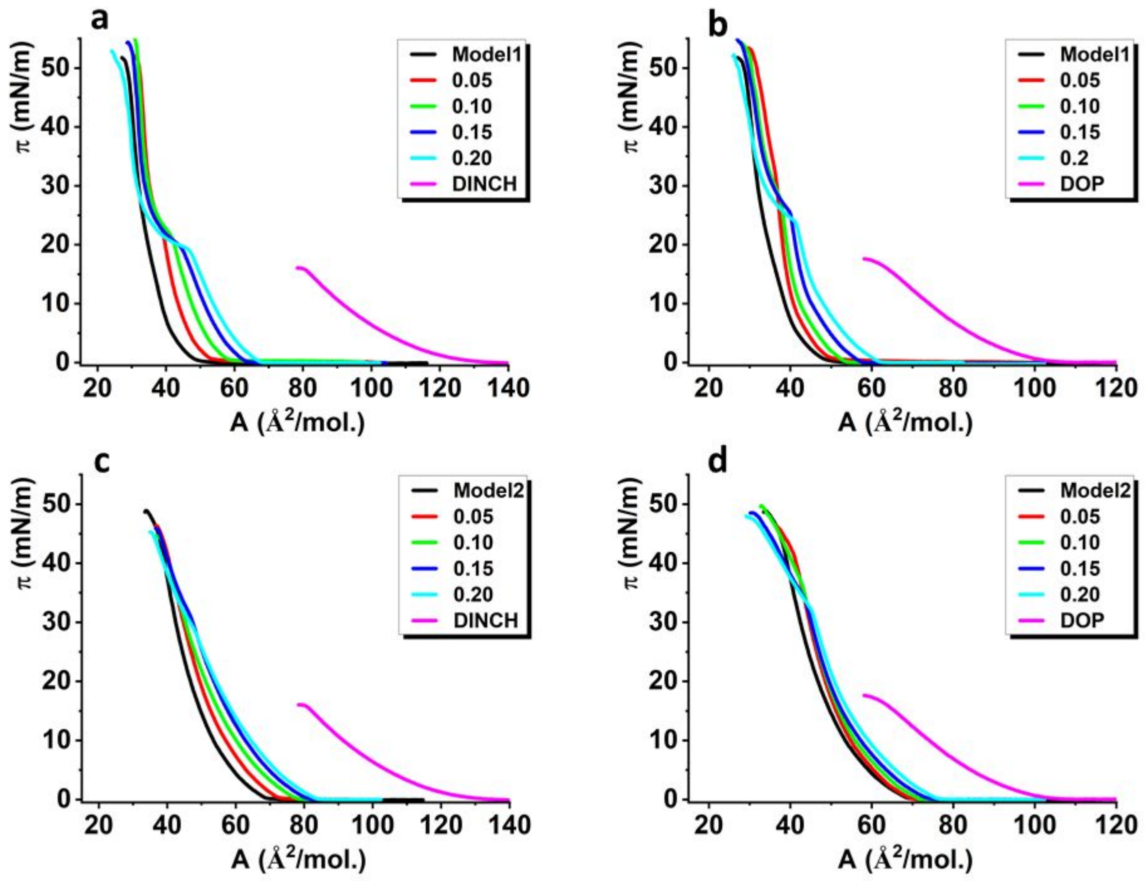

Fig. S7. π-A isotherms for the systems: a) Mod1_DINCH, b) Mod1_DOP, c) Mod2_DINCH, d) Mod2_DOP.

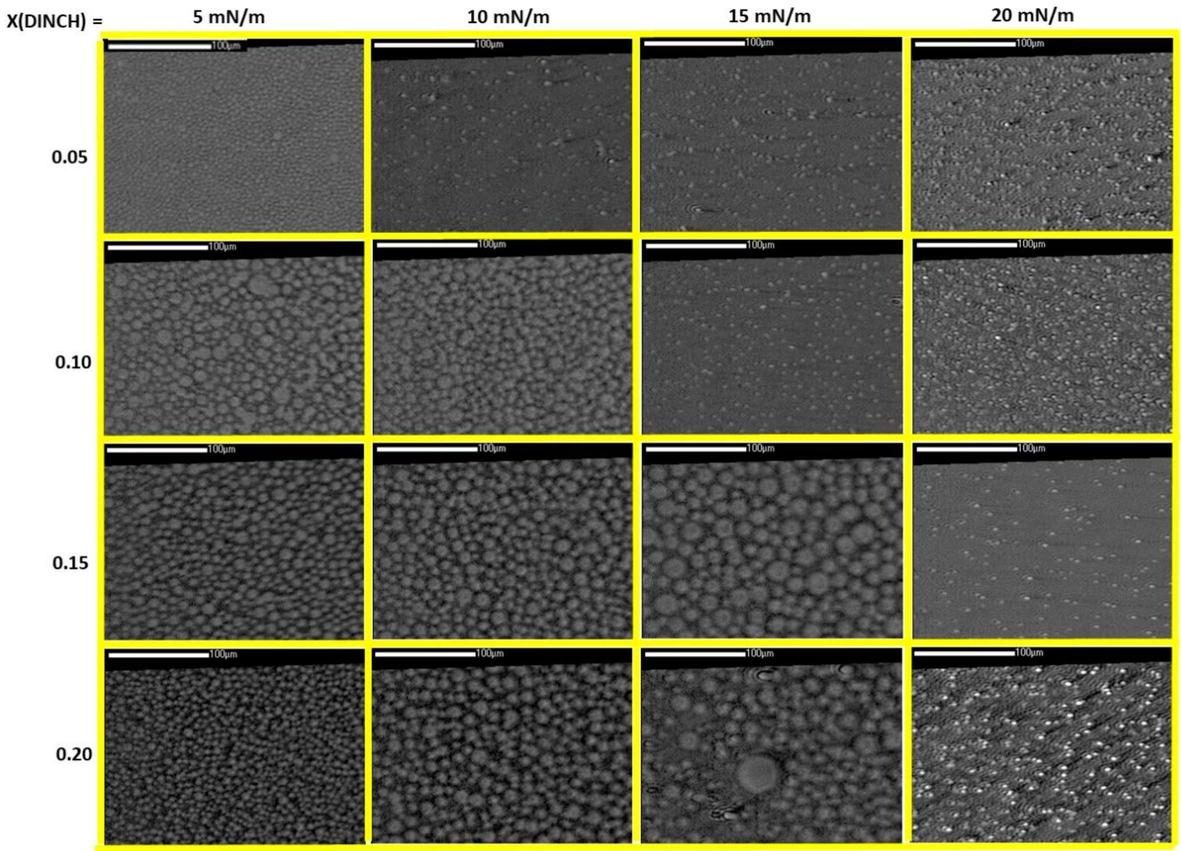

Fig. S8A. Selected BAM images for the system Mod1_DINCH

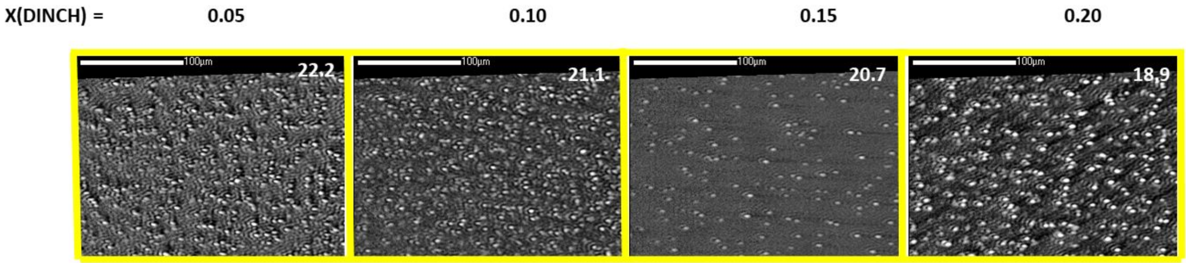

Fig. S8B. Aggregation in the system Mod1_DINCH. The numbers in the images indicate the surface pressure at which 3D aggregates appeared.

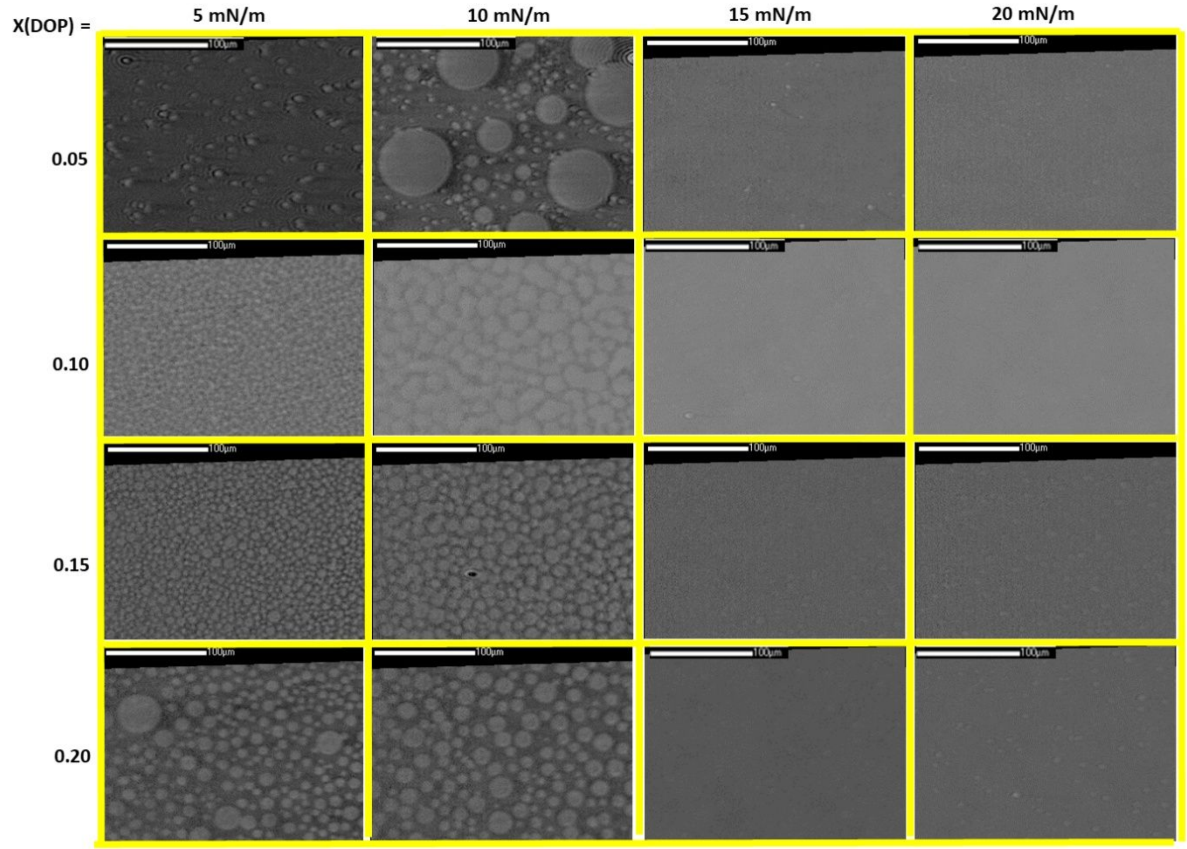

Fig. S9A. Selected BAM images for the system Mod1_DOP

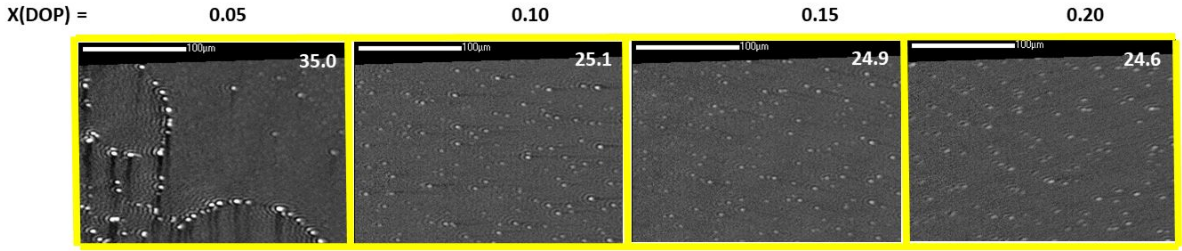

Fig. S9B. Aggregation in the system Mod1_DOP. The numbers in the images indicate the surface pressure at which 3D aggregates appeared.

V. Characterization of the model membranes enriched in DIDP, DDP, and DTP

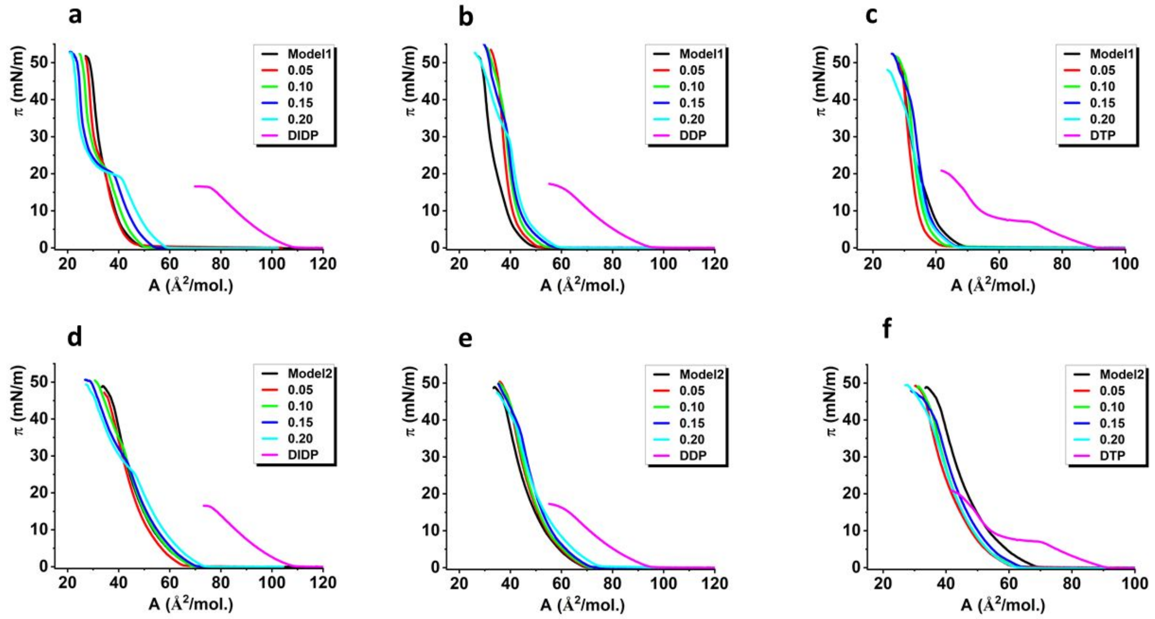

Fig. S10. Π-A isotherms for the systems: a) Mod1_DIDP, b) Mod1_DDP, c) Mod1_DTP, d) Mod2_DIDP, e) Mod2_DDP, f) Mod2_DTP

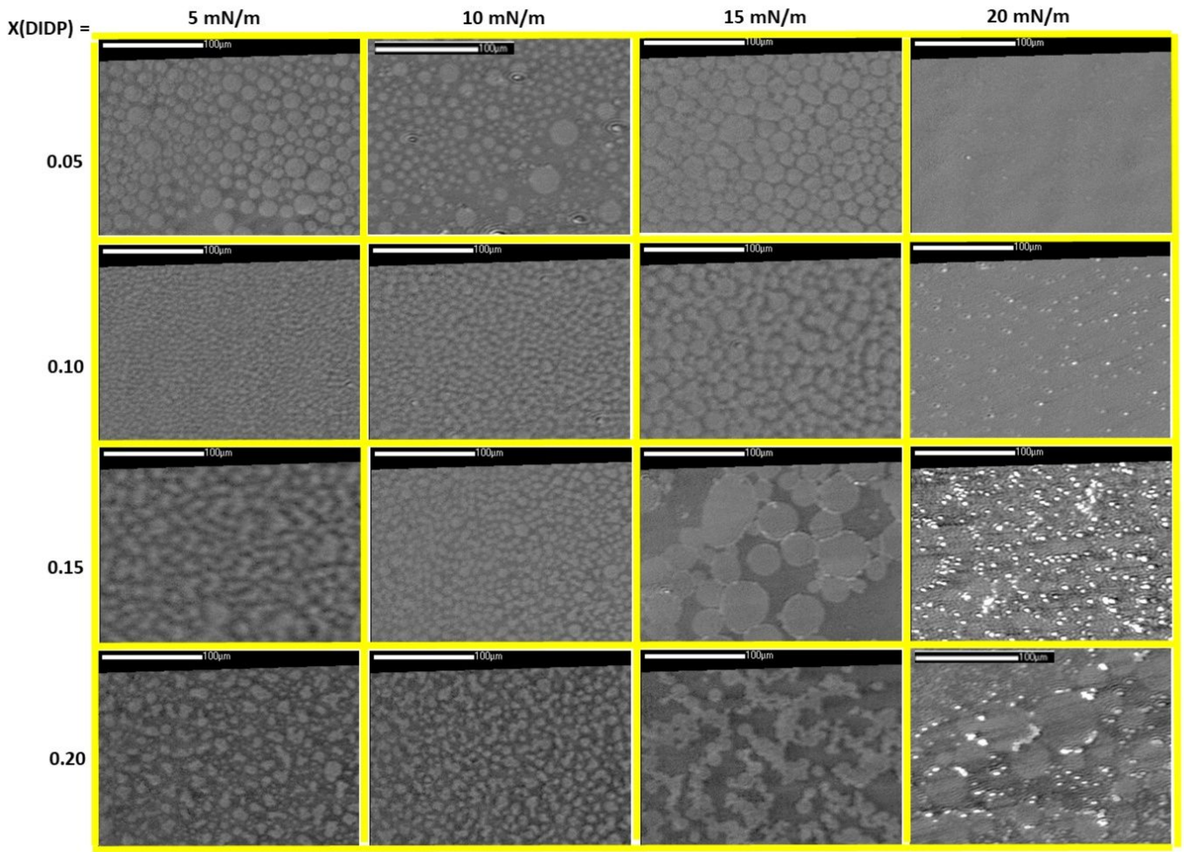

Fig. S11A. Selected BAM images for the system Mod1_DIDP.

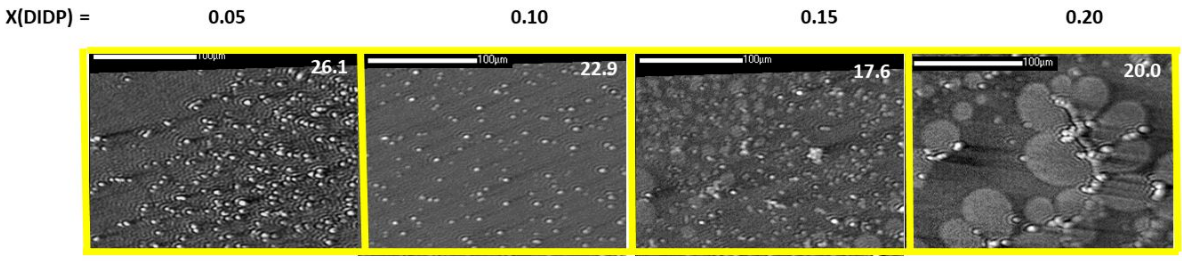

Fig. S11B. Aggregation in the system Mod1_DIDP. The numbers in the images indicate the surface pressure at which 3D aggregates appeared.

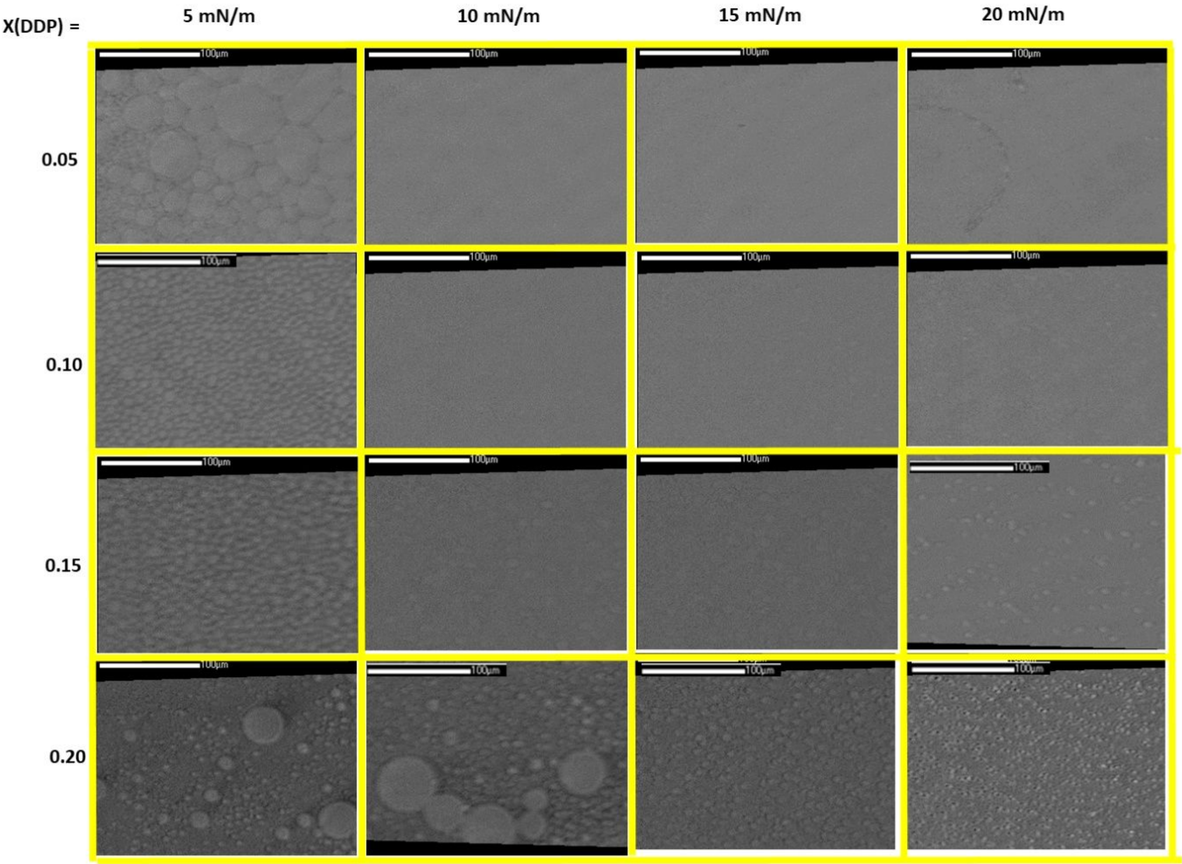

Fig. S12A. Selected BAM images for the system Mod1_DDP.

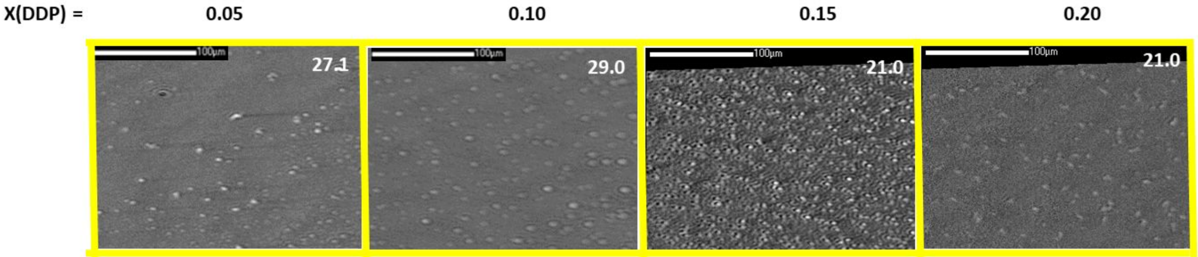

Fig. S12B. Aggregation in the system Mod1_DDP. The numbers in the images indicate the surface pressure at which 3D aggregates appeared.

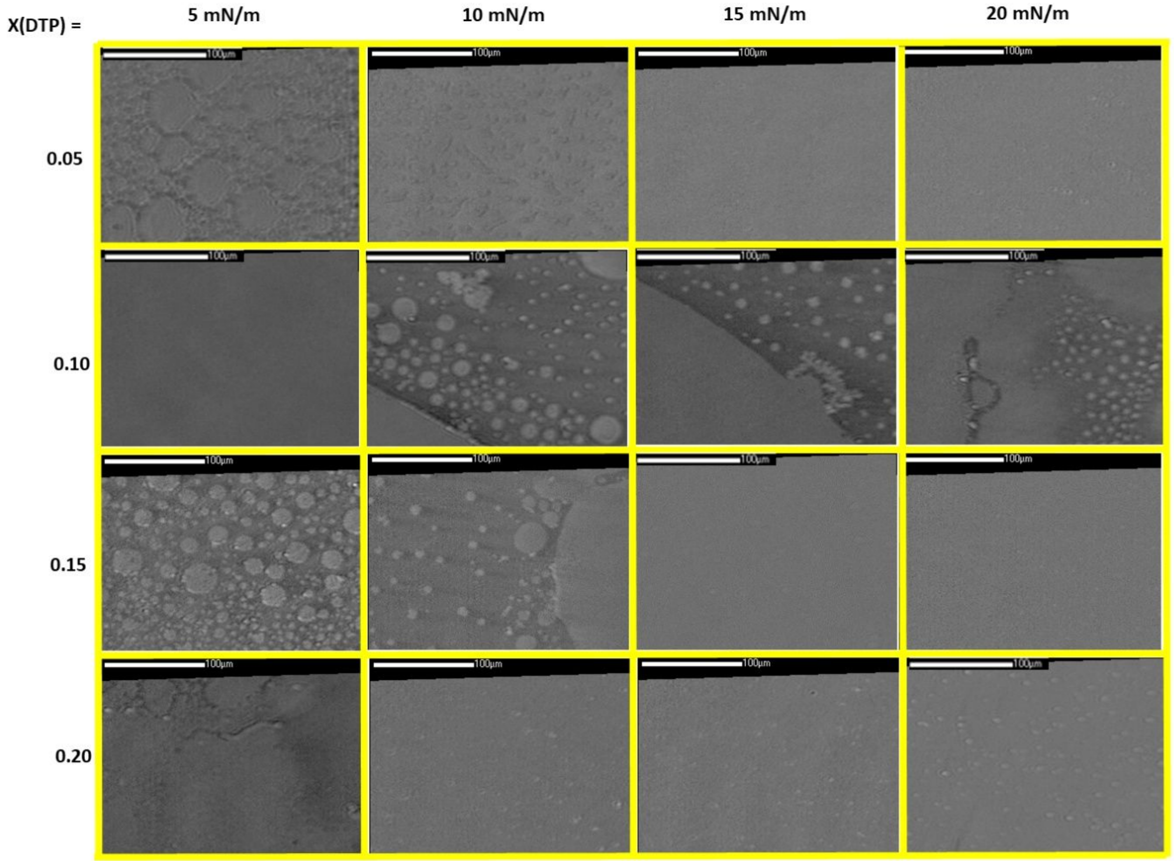

Fig. S13A. Selected BAM images for the system Mod1_DTP.

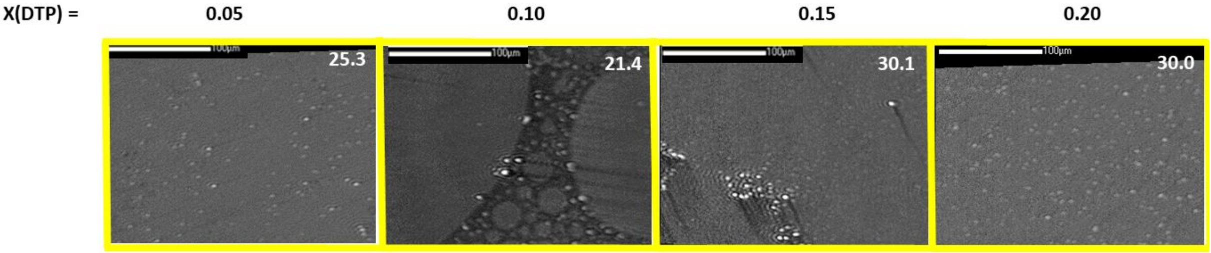

Fig. S13B. Aggregation in the system Mod1_DTP. The numbers in the images indicate the surface pressure at which 3D aggregates appeared.
